# HAND factors regulate cardiac lineage commitment and differentiation from human pluripotent stem cells

**DOI:** 10.1186/s13287-024-03649-9

**Published:** 2024-02-05

**Authors:** Huixin Guo, Chengwen Hang, Bowen Lin, Zheyi Lin, Hui Xiong, Mingshuai Zhang, Renhong Lu, Junyang Liu, Dan Shi, Duanyang Xie, Yi Liu, Dandan Liang, Jian Yang, Yi-Han Chen

**Affiliations:** 1 Department of Cardiology, The Second Hospital of Shanxi Medical University, Taiyuan, 030001 China; 2 State Key Laboratory of Cardiology, Shanghai East Hospital, Tongji University School of Medicine, Shanghai, 200120 China; 3 Shanghai Arrhythmia Research Center, Shanghai East Hospital, Tongji University School of Medicine, Shanghai, 200120 China; 4 Department of Cardiology, Shanghai East Hospital, Tongji University School of Medicine, Shanghai, 200120 China; 5 Shanghai Frontiers Center of Nanocatalytic Medicine, Shanghai, 200092 China; 6 Department of Pathology and Pathophysiology, Tongji University School of Medicine, Shanghai, 200092 China; 7 Department of Cell Biology, Tongji University School of Medicine, Shanghai, 200092 China; 8 Research Units of Origin and Regulation of Heart Rhythm, Chinese Academy of Medical Sciences, Shanghai, 200092 China

**Keywords:** *HAND1*, *HAND2*, Human pluripotent stem cells, Cardiac lineage commitment, Cardiomyocyte differentiation, *TBX5*

## Abstract

**Background:**

Transcription factors HAND1 and HAND2 (HAND1/2) play significant roles in cardiac organogenesis. Abnormal expression and deficiency of *HAND1*/*2* result in severe cardiac defects. However, the function and mechanism of *HAND1*/*2* in regulating human early cardiac lineage commitment and differentiation are still unclear.

**Methods:**

With *NKX2.5*^*eGFP*^ H9 human embryonic stem cells (hESCs), we established single and double knockout cell lines for *HAND1* and *HAND2*, respectively, whose cardiomyocyte differentiation efficiency could be monitored by assessing NKX2.5-eGFP^+^ cells with flow cytometry. The expression of specific markers for heart fields and cardiomyocyte subtypes was examined by quantitative PCR, western blot and immunofluorescence staining. Microelectrode array and whole-cell patch clamp were performed to determine the electrophysiological characteristics of differentiated cardiomyocytes. The transcriptomic changes of *HAND* knockout cells were revealed by RNA sequencing. The HAND1/2 target genes were identified and validated experimentally by integrating with HAND1/2 chromatin immunoprecipitation sequencing data.

**Results:**

Either *HAND1* or *HAND2* knockout did not affect the cardiomyocyte differentiation kinetics, whereas depletion of *HAND1*/*2* resulted in delayed differentiation onset. *HAND1* knockout biased cardiac mesoderm toward second heart field progenitors at the expense of first heart field progenitors, leading to increased expression of atrial and outflow tract cardiomyocyte markers, which was further confirmed by the appearance of atrial-like action potentials. By contrast, *HAND2* knockout cardiomyocytes had reduced expression of atrial cardiomyocyte markers and displayed ventricular-like action potentials. *HAND1*/*2*-deficient hESCs were more inclined to second heart field lineage and its derived cardiomyocytes with atrial-like action potentials than *HAND1* single knockout during differentiation. Further mechanistic investigations suggested *TBX5* as one of the downstream targets of HAND1/2, whose overexpression partially restored the abnormal cardiomyocyte differentiation in *HAND1*/*2*-deficient hESCs.

**Conclusions:**

*HAND1*/*2* have specific and redundant roles in cardiac lineage commitment and differentiation. These findings not only reveal the essential function of *HAND1/2* in cardiac organogenesis, but also provide important information on the pathogenesis of *HAND1*/*2* deficiency-related congenital heart diseases, which could potentially lead to new therapeutic strategies.

**Supplementary Information:**

The online version contains supplementary material available at 10.1186/s13287-024-03649-9.

## Background

Normal cardiac morphogenesis relies on the precise regulation of numerous transcription factors (TFs) [1]. Mutations in these TFs are known to cause common cardiac defects [2]. Congenital heart diseases (CHDs) are the most prevalent congenital defects, with a morbidity rate of approximately 1% [3], in which 25% of infants require intervention within the first year after birth [4]. Despite advances in medication and surgical procedures, CHDs continue to be the leading cause of death in individuals with congenital defects [5]. Furthermore, survivors of CHDs often experience cardiac comorbidities, reduced quality of life and increased burden [6]. Due to the characteristic segmental development of the heart, CHDs primarily affect a single chamber or a specific part of the heart [7].

As the chamber-specific TFs, HAND1 and HAND2 (HAND1/2) play crucial roles in cardiac morphogenesis [8]. They are members of the basic helix–loop–helix (bHLH) family, functioning through DNA-binding, protein–protein interaction and reprograming the enhancer/promoter connectome [9–11]. Currently, it has been accepted that the cardiac lineage is determined at primitive streak and mesoderm stages during embryo development [12–14]. Then, the cardiac mesodermal cells migrate and form cardiac crescent to generate the first heart field (FHF) and second heart field (SHF) progenitors, which produce different subtypes of cardiomyocytes [15]. The FHF progenitors are characterized by the expression of *HAND1*, *TBX5* and *HCN4* [16–18], and ultimately differentiate into left ventricular cardiomyocytes. The SHF is further classified as anterior SHF (aSHF) and posterior SHF (pSHF). The aSHF progenitors are identified by the markers *TBX1*, *SIX1* and *FOXC2* [19, 20], and primarily give rise to right ventricular and outflow tract (OFT) cardiomyocytes while the pSHF progenitors are distinguished by *NR2F2*, *HOXA1*, *HOXB1* and *ALDH1A2* expression [19, 20], and mainly develop into atrial cardiomyocytes. In mouse, the single-cell transcriptome of embryos showed that *Hand1* was first detected in nascent mesoderm at E6.5-E6.75 [21]. At E7.75, *Hand1* was expressed in cardiac crescent which contributed to a subset of left ventricular cardiomyocytes later [22–24]. *Hand2* was first detected in nascent mesoderm at E7.0 [21], then, together with *Hand1,* expressed in cardiac crescent and played vital roles in the patterning of heart fields [15, 25]. Knockout of *Hand1* led to left ventricular hypoplasia, interventricular septal defects and cardiac conduction system defects [26, 27]. *Hand2* was required for the survival and differentiation of SHF progenitors and its deficiency resulted in right ventricular defects [25, 28–30]. Besides, mice with *Hand1* and *Hand2* double knockout were embryonically lethal, displaying a single ventricle and a common atrium [27].

Due to the lack of human embryo data, the expression profile of human *HAND* was not as clear as that of the mouse. Currently available early human embryo single-cell sequencing data showed that *HAND1* and *HAND2* were detected in mesoderm of CS7 (Carnegie stage 7) embryo [31], which is equivalent to mouse E7.0 [32], and expressed in lateral plate mesoderm of human CS10-CS14 embryos [33]. In human heart, *HAND1* was specifically expressed in left ventricular cardiomyocytes, which originate from the FHF [34, 35], while *HAND2* showed high expression in atrial cardiomyocytes, which are the progeny of the SHF [35, 36]. Concomitantly, during cardiomyocyte differentiation from human pluripotent stem cells (hPSCs), *HAND1* was highly expressed in FHF progenitors and ventricular cardiomyocytes, while *HAND2* was upregulated in differentiated atrial cardiomyocytes [19, 37]. Furthermore, pathogenic mutations of *HAND1* were related to left ventricular hypoplasia and ventricular septum defects [38–40], while *HAND2* deficiency contributed to ventricular septum defects and double outlet right ventricle [41]. In severe CHDs, such as tetralogy of Fallot, mutations of *HAND1*/*2* have been identified as well [42, 43]. Nevertheless, the roles of *HAND* in human early cardiac lineage commitment and differentiation are not completely clarified and need further investigation.

The pathogenicity of *Hand* in mouse is not the same as human *HAND1*/*2* mutation related CHDs. In addition, the embryo development and gene expression between primates and rodents are different [35, 44], which restricts the application of mouse model to investigate *HAND* function in human heart development and related CHDs. hPSCs have been widely selected as alternative for animal models in studying human diseases in vitro. With the scalability of cardiomyocyte differentiation [45] and the ability to mimic the in vivo heart development process, hPSCs have broad applications such as cardiac disease models, cardiac regeneration and drug screening [46–48], especially in cardiac lineage development [49, 50].

Here, we utilized the *NKX2.5*^*eGFP*^ H9 human embryonic stem cells (hESCs) along with the clustered regularly interspaced short palindromic repeats (CRISPR)/Cas9 technology to establish *HAND1*/*2* single and double knockout cell lines. With an in vitro cardiomyocyte differentiation system, we systematically investigated the effects of *HAND1* and *HAND2* deficiency on cardiac lineage commitment and differentiation. We characterized the unique and redundant roles of *HAND1* and *HAND2* and verified *TBX5* as one of the key targets in *HAND1*/*2* gene regulatory network during heart development.

## Methods

### Human embryonic stem cell culture

Human embryonic stem cell H9 (hESC-H9) based *NKX2.5* reporter cell line (*NKX2.5*^*eGFP*^ H9) was purchased from Shanghai model organism Co., Ltd. *NKX2.5*^*eGFP*^ H9 and its derived hESC lines were cultured in Matrigel (Corning, USA) coated cell culture plates with PSCeasy culture medium (Cellapy, China) at 37 °C, 5% CO_2_. When reaching 80–90% confluence, the cells were dissociated with human multipotent stem cell digestive solution (Cellapy), passaged at 1:3 to 1:6, and cultured in PSCeasy medium with ROCK inhibitor Y-27632 (10 μM) for 24 h. Then, the medium was replenished every day without Y-27632.

### Cardiomyocyte differentiation and culture

The cardiomyocyte differentiation protocol was conducted by modulating canonical Wnt signaling with some modifications [51, 52]. When *NKX2.5*^*eGFP*^ H9 cells grew to 80–90% confluence, the cells were dissociated with Accutase (Sigma-Aldrich, USA) and resuspended in PSCeasy with Y-27632. Accurate cell counting was necessary to achieve stable differentiation efficiency. 1.5–1.8 × 10^5^ cells (*NKX2.5*^*eGFP*^ H9 and *HAND* single knockout cell lines) were plated in Geltrex (Gibco, USA) coated 12-well plates for the following differentiation. For *HAND* double knockout cell line, the differentiation started with 1.5–1.6 × 10^5^ cells per 12-well.

On the second day after seeding, the medium was switched to N2B27 (100 mL DMEM/F12, 100 mL Neurobasal with 1 mL N2 and 2 mL B27) [53, 54] with 3 μM CHIR99021 (Selleck, USA) to activate WNT signaling for 48 h (days 0–2). Then, the medium was changed to RPMI1640/B27 minus insulin (RPMI/B27-) for 24 h (day 2–3). At day 3, 2 μM Wnt-C59 (MCE, USA) was added into RPMI/B27- to inhibit WNT signaling for 48 h (days 3–5), then the cells were cultured in RPMI/B27- for 2 days (days 5–7). At day 7, the medium was changed to RPMI/B27 for continuous differentiation. 0.5 mM Vitamin C (Vc) was added to the differentiation medium during days 0–7. The eGFP fluorescence and beating cardiomyocytes were monitored under fluorescence microscope.

To culture hESCs-derived cardiomyocytes, the cells were dissociated with human cardiomyocytes digestive solution I (Cellapy) for 13–15 min and digestive solution II (Cellapy) for 20–25 min at 37 °C. Cells were gently detached and centrifuged at 1200 rpm for 5 min. The cells were resuspended in RPMI/B27 with Y-27632 and replated for the following experiments. Medium was switched to RPMI/B27 after 48 h and then replenished every other day.

### Vector construction

Human *HAND1* or *HAND2* cDNA was cloned into PBCAG transposon under the control of the CAG promotor using homologous recombination to generate PBCAG-*HAND1* and PBCAG-*HAND2* vectors. Human *TBX5* cDNA was cloned into PBTRE transposon under the control of doxycycline (DOX) inducible Tet response element using homologous recombination to generate PBTRE-*TBX5* vector.

To construct *TBX5* promoter driven luciferase expression vector, the sequence of *TBX5* promoter (− 2 kb ~  + 100 bp) was downloaded from the website (https://pubmed.ncbi.nlm.nih.gov/) and amplified from the genome of H9 hESCs with PCR. The purified PCR product was ligated to the restriction enzyme digested pGL3-basic Luciferase vector fragment (Promega, WI, USA) by homologous recombination to construct the pGL3-*TBX5-Luciferase* vector. The primers used are listed in Additional file 1: Table S1.

### Generation of *HAND* gene knockout (KO) cell line

Single guide RNA (sgRNA) was designed on the website (http://crispor.tefor.net/) for genome editing. *HAND1*-sgRNA (5′AGCGCGAGGCCGGACCGAAG3′) and *HAND2*-sgRNA (5′GGACCACTCCCATTACGGGG3′) targeting exon 1 of *HAND1* and *HAND2*, respectively, were used for constructing *HAND* gene KO cell lines. The sgRNA was cloned into pGL3-U6-PGK-*Puromycin* vector. 1 × 10^6^ *NKX2.5*^*eGFP*^ H9 cells were transfected with 1 μg sgRNA and 2 μg spCas9 by LONZA P3 primary cell 4D-Nucleofector LV KIT according to the manufacturer’s protocol. After transfection, the cells were selected with 0.3 μg/mL puromycin for 3–7 days, then replated as single cells to form clones. During days 7–10, clones were picked and expanded for genotyping. Genomic PCR and Sanger sequencing were used to clarify the details of editing. Primers are listed in Additional file 1: Table S1.

### Generation of *TBX5* overexpressing cell lines

The *HAND1*/*2*-double-KO cell line was electro-transfected with 1 μg PBTRE-*TBX5*, 1 μg PBEF1α-*Tet3G*, and 1 μg HyPBase. The cells were selected with 0.3 μg/mL puromycin for 7 days and clones were picked. Genomic PCR was performed to identify positive clones. The expression of *TBX5* transgene under 1 μg/mL DOX induction was confirmed by western blot.

### In vitro* embryoid body (EB) differentiation*

EB differentiation was conducted as previously described [55]. 1 × 10^6^ cells in PSCeasy with Y-27632 were plated into 6-cm petri dish on a shaker, at 60 rpm, to form EBs. After two days, when the size of EBs reached about 200 μm in diameter, the medium was switched to DMEM/F12 with 20% knockout serum replacement (KSR, Gibco, USA), then replenished every other day. After 8 days, the EBs were plated into 0.2% gelatin-coated plates for further culture. At day 15, EBs were collected for analysis.

### Flow cytometry analysis and fluorescence-activated cell sorting

For flow cytometry analysis of SSEA-4 or MYL2 expression, hESCs or cardiomyocytes were fixed with 4% paraformaldehyde (PFA) for 15 min at room temperature (RT). Cells were permeabilized with 0.3% PBSTr (Triton-X100) for 15 min, RT (this step was not needed for SSEA-4). Then cells were blocked with 3% bovine serum albumin (BSA) in PBS for 30 min. After that, the cells were incubated with anti-SSEA-4 antibody (Santa Cruz, sc59368, 1:200) or anti-MYL2 antibody (Abcam, ab79935, 1:100) at 4 °C overnight. The next day, the cells were washed with PBS three times and then incubated with fluorescence-conjugated secondary antibody for 1 h, RT. After washing with PBS three times, the cells were harvested for analysis.

To monitor cardiomyocyte differentiation, at different time points, the cells were dissociated into single cells and washed with PBS twice, then filtered with 40 μm strainer. The percentage of NKX2.5-eGFP^+^ cells was determined by flow cytometry. FlowJo 10 and NovoExpress software were used for data analysis.

For cell sorting, at differentiation day 7, the cells were dissociated and resuspended in RPMI/B27. After being filtered with 40 μm strainer, the NKX2.5-eGFP^+^ cells were sorted by Beckman Coulter MoFlo Astrios EQ for subsequent analysis.

### Dual-Luciferase assay

Dual-Luciferase assay was performed with HEK 293T cells to verify *TBX5* as the target of HAND1/2. The cells were cultured in DMEM with 10% FBS (Gibco, USA) in 6-well plates. When reaching 40–50% confluence, the cells were transfected with 1 μg pGL3-*TBX5*-*Luciferase*, 0.1 μg *Renilla* plasmid and 1 μg PBCAG-*HAND1* or PBCAG-*HAND2* using Lipofectamine 3000 transfection reagent (Invitrogen, USA). Samples were harvested and analyzed 48 h after transfection, according to the manufacturer’s protocol (Promega).

### RNA extraction, reverse transcription and Quantitative PCR (qPCR)

Cells were washed twice with PBS and lysed in Trizol (Takara, Japan). After chloroform extraction, the supernatant was precipitated with isopropanol, washed with 75% ethanol and dissolved with RNase-free water. The RNA concentration was measured with a Spectrophotometer (NanoDrop Technologies, Inc., DE, USA). The reverse transcription was carried out following the instruction of HiScript III 1st Strand cDNA Synthesis Kit (Vazyme, China). qPCR was performed using Taq Pro Universal SYBR qPCR Master Mix with specific primers on ABI QuantStudio™ 6 Flex (Thermofisher). *GAPDH* expression was used to normalize the gene expression. The gene expression between different groups was compared with ΔΔCT method. The primers used are listed in Additional file 1: Table S1.

### Western blot

Cells were dissociated and centrifuged at 1200 rpm for 5 min, then lysed in RIPA buffer with protease inhibitors. After incubating on ice for 30 min, the lysis was centrifuged at 12,000*g* for 15 min, the supernatant was collected and the protein concentration was measured with BCA methods (Beyotime Biotechnology, China). The samples were boiled for 5 min at 100 °C. NuPAGE 10% Bis–Tris gel was used for electrophoresis under 100 mv for 15 min and then 170 mV for 40 min. The protein was transferred onto PVDF membrane. After blocking with 5% non-fat milk, the membrane was incubated with primary antibodies overnight at 4 °C. The next day, after washing, the membrane was incubated with Alexa Fluor conjugated secondary antibodies for 1 h, RT. The images were captured with the Odyssey system. Primary antibodies used were: anti-GAPDH (Proteintech, 60004-1-Ig, 1:5000), anti-HAND1 (Abclonal, A9855, 1:500), anti-HAND2 (Abcam, ab200040, 1:1000), anti-CX43 (Sigma-Aldrich, C6219, 1:3000), anti-MYL2 (Proteintech, 10906-1-AP, 1:1000), anti-NR2F2 (Cell Signaling Technology, 6434, 1:1000) and anti-SCN5A (Proteintech, 23016-1-AP, 1:1000).

### Immunofluorescence staining

Cells were fixed with 4% PFA for 15 min, RT, washed with PBS three times, then permeabilized with 0.3% PBSTr (Triton-X100) for 15 min. Permeabilization is not required for cell surface antigens. After blocking in 3% BSA for 30 min, the cells were incubated with primary antibodies at 4 °C, overnight. The next day, after washing with 0.1% PBST (Tween) three times, the cells were incubated with fluorescence-conjugated secondary antibodies (Abcam, 1:200) for 1 h, RT, then washed with 0.1% PBST three times. The nuclei were stained with DAPI (Sigma-Aldrich, 1:10000). Images were captured with fluorescence microscope (Leica, Germany). The primary antibodies used were: anti-SSEA-4 (Santa Cruz, sc59368, 1:200), anti-OCT4 (Abcam, ab181557, 1:200), anti-TBXT (R&D systems, AF2085, 1:200), anti-CX43 (Sigma-Aldrich, C6219, 1:400), and anti-NR2F2 (Santa Cruz, sc393481, 1:100).

### Microelectrode array (MEA) analysis

Cardiomyocytes from differentiation day 30 were dissociated into single cells and counted. 3 × 10^4^ Cells were seeded onto Geltrex coated 24-well CytoView MEA plate. Field potential recording was conducted when the cells began to beat. Local extracellular action potential (LEAP) induction was used to simulate action potential. Corrected action potential duration (APD) was calculated using Bazett’s formula [56]. Data were analyzed by Cardiac Analysis Tool, AxionDataExportTool and Igor.

### Whole-cell patch clamp

Whole-cell patch clamp was performed for the electrophysiological characteristics of hESCs-derived cardiomyocytes. 1 × 10^4^ cells were plated into growth-factor-reduced Matrigel (Corning) coated 3.5-cm dish. Patch clamp was conducted between days 2–6 after plating. The tip resistance of Borosilicate glass microelectrodes was 2–3 MΩ. The pipette solution was composed of 140 mM KCl, 10 mM EGTA, 5 mM glucose, 3 mM MgATP and 10 mM HEPES, adjusting pH to 7.2 with KOH. The bath solution was: 140 mM NaCl, 5.4 mM KCl, 1.8 mM CaCl_2_, 1 mM MgCl_2_, 1.2 mM KH_2_PO_4_, 5.5 mM glucose and 5 mM HEPES, adjusting pH to 7.4 with NaOH and the solution was oxygenated for at least 30 min before use. Spontaneous and paced action potential were recorded with EPC-10 amplifier (HEKA, Germany). The data were analyzed with Minianalysis and Clampfit.

### RNA-sequencing (RNA-seq) library preparation and sequencing

NKX2.5-eGFP^+^ cells were sorted at differentiation day 7. Total RNA was extracted using the RNA extraction kit (Sigma-Aldrich). One microgram of total RNA was used in mRNA capture by NEBNext PolyA mRNA Magnetic Isolation Module (New England Biolabs, MA, USA). The RNA-seq libraries were constructed according to the instruction of NEB Next Ultra Directional RNA Library Prep Kit for Illumina (New England Biolabs, MA, USA). RNA-seq libraries were then sequenced as 150-bp paired-end reads on an Illumina NovaSeq 6000 platform. The sequencing was performed by Shanghai Genefund Biotech Co., Ltd.

### RNA-seq data processing

Raw paired-end RNA-seq reads were trimmed to remove adapters by Trim Galore! (version 0.6.4_dev). The clean reads were aligned to the human genome (assembly GRCh38) using Hisat2 (version 2.2.1) [57]. The counts per gene were quantified to the exon level (-t exon) by featureCounts (version 2.0.1) [58]. Reads were normalized to transcripts per million (TPM) for visualization by a compiled R script (R version 4.0.2). Principal component analysis (PCA) was performed through the irlba (version 2.3.3). Differential expression analysis was performed using DESeq2 (version 1.28.1) [59], and the genes with $|{\text{fold}}\;{\text{change }}\left( {{\text{FC}}} \right)|$> 2 and adjusted *P* value < 0.05 were considered as differentially expressed genes (DEGs). The fuzzy c-means clustering algorithm identified eight distinct gene expression clusters by Mfuzz (version 2.48.0) [60]. Gene ontology (GO) and Kyoto Encyclopedia of Genes and Genomes (KEGG, https://www.genome.jp/kegg/) analyses were performed using clusterProfiler (version 3.16.0) [61]. Adjusted *P* value < 0.05 was considered significant in the enrichment analysis.

### Statistical analysis

Data were displayed as mean ± SEM. Statistical analysis was conducted using Prism 8 (GraphPad, Boston, MA). Unpaired 2-tailed Student *t* test and one-way ANOVA were used for the comparison of groups. Statistical significance was defined as *P* value < 0.05.

## Results

### Establishment of *HAND1*-KO, *HAND2*-KO and *HAND1*/*2*-double-KO (dKO) hESC lines

Previous studies have verified that the introduction of enhanced GFP (*eGFP*) into *NKX2.5* locus could be utilized to isolate and characterize cardiomyocytes differentiated from hESCs [62]. We thus applied *NKX2.5*^*eGFP*^ H9 [designated as wild type (WT) hereafter], which was generated by inserting *eGFP* into the start codon of *NKX2.5* (Additional file 1: Fig. S1A), to investigate the function of *HAND1* and *HAND2* in cardiomyocyte differentiation.

**Fig. 1:**
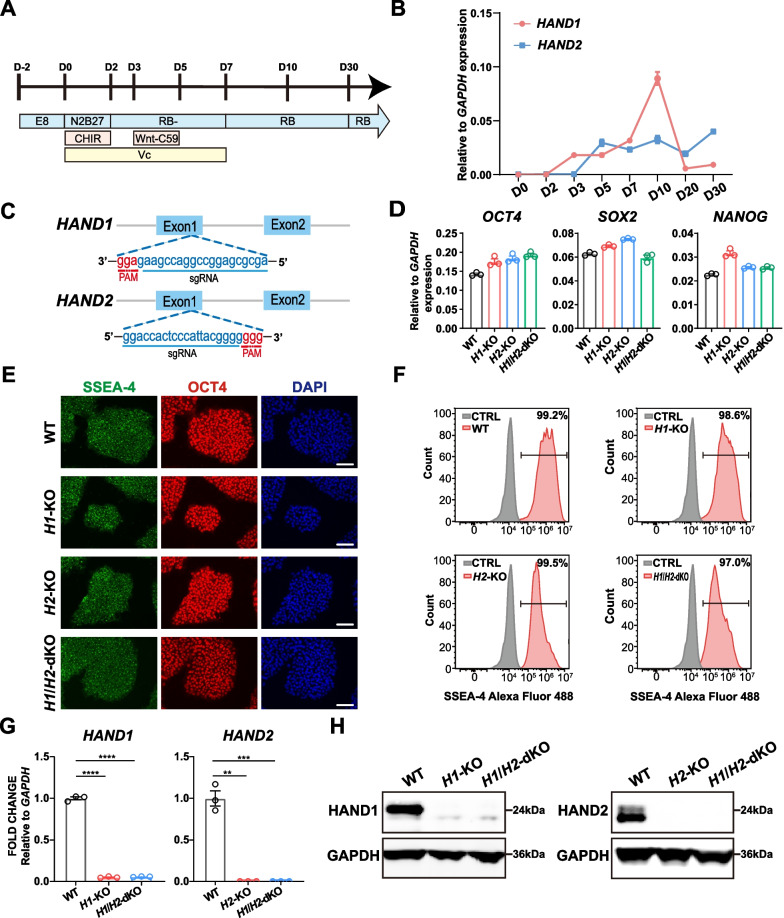
Establishment of *H1*-KO, *H2*-KO and *H1*/*H2*-dKO hESC lines. **A** Schematic diagram of cardiomyocyte differentiation protocol. Mesoderm stage was at day 2. Cardiac mesoderm stage was at day 3. Cardiac progenitor stage was at day 5. Cardiomyocytes started beating at day 7. RB-: RPMI/B27-, RB: RPMI/B27, CHIR: CHIR99021, Vc: Vitamin C. **B** Temporal expression of *HAND1* and *HAND2* during cardiomyocyte differentiation. Relative to *GAPDH* expression (n = 3). **C** Schematic diagram of CRISPR/Cas9 mediated *HAND1*/*2* genome editing. PAM: protospacer adjacent motif. **D** Expression of *OCT4*, *SOX2* and *NANOG* in WT and *HAND* KO hESC lines. Relative to *GAPDH* expression (n = 3). **E** Immunofluorescence staining of SSEA-4 and OCT4 in WT and *HAND* KO hESC lines. Scale bar = 100 μm. **F** Flow cytometry analysis of SSEA-4^+^ cells in WT and *HAND* KO hESC lines. CTRL represented the negative control with secondary antibody incubation only. **G** qPCR analysis of *HAND1* and *HAND2* in WT and *HAND* KO hESC lines-derived day 10 cardiomyocytes. Relative to *GAPDH* expression (n = 3). One-way ANOVA. **H** Western blot analysis of HAND1 and HAND2 expression in WT and *HAND* KO hESC lines-derived differentiation day 10 cardiomyocytes. GAPDH served as loading control. Corresponding uncropped full-length gels and blots are presented in Additional file 8: Fig. S7. **p < 0.01, ***p < 0.001, ****p < 0.0001

We then employed a modified cardiomyocyte differentiation protocol by modulating canonical Wnt signaling [51]. hESCs were treated with sequential Wnt activator and inhibitor and concomitant use of Vc to promote cardiomyocyte differentiation [52] (Fig. 1A). At day 7, the NKX2.5-eGFP^+^ cells emerged and the differentiated cardiomyocytes started to beat (Additional file 2: Supplementary Video 1). The differentiation efficiency could reach more than 80% during differentiation days 10–30 (Additional file 1: Fig. S1B), indicating the robustness of this protocol.

We then examined the expression level of *HAND1/2* at different time points during the differentiation. *HAND1* began to be expressed on day 3, at the cardiac mesoderm stage [63, 64], and continued to increase to day 10, then maintained at a low-level during days 20–30, while *HAND2* was first detected on day 5, at the cardiac progenitor stage [63, 64], and expressed at a relatively stable level during the differentiation (Fig. 1B). Expression of *HAND1* preceded that of *HAND2* during our monolayer cardiomyocyte differentiation, consistent with previous study using EB differentiation protocol [37].

Next, we knocked out *HAND1*, *HAND2* in *NKX2.5*^*eGFP*^ H9 cells to establish *HAND1*-KO, *HAND2*-KO and *HAND1*/*2*-dKO cell lines (designated as *H1*-KO, *H2*-KO and *H1*/*H2*-dKO hereafter) with CRISPR/Cas9 technology. Exon 1 of *HAND1*/*2* was targeted to generate *H1*-KO or *H2*-KO cell lines, respectively (Fig. 1C). For *H1*/*H2*-dKO cell lines, the sgRNAs of *HAND1* and *HAND2* were transfected into *NKX2.5*^*eGFP*^ H9 cells with spCas9 simultaneously. Homozygous KO cell lines of *HAND1* or/and *HAND2* were selected for further analysis. We obtained at least two monoclonal clones with different gene editing for each KO cell line. Two independent clones from each line were selected for subsequent studies (Additional file 1: Fig. S1C). PCR and Sanger sequencing confirmed that the selected cell lines had no predicted off-targets (Additional file 1: Fig. S1D). Besides, the KO cell lines showed normal karyotype with 46, XX (Additional file 1: Fig. S1E).

To compare the stemness of *HAND* KO cell lines with WT, we determined the expression of pluripotency genes, *OCT4*, *SOX2* and *NANOG* [65], which was comparable among WT and KO cell lines (Fig. 1D). Also, immunofluorescence staining of pluripotent cell surface marker SSEA-4 [65] and OCT4 showed no difference (Fig. 1E). The SSEA-4^+^ cells were consistently above 95% in all cell lines (Fig. 1F). The expression of tridermic markers was low, similar to WT, suggesting that *HAND* KO cell lines did not differentiate significantly toward the three germlayers in stem cell culture (Additional file 1: Fig. S1F). The pluripotency of KO cell lines was further demonstrated by the expression of endoderm (AFP), mesoderm (α-SMA) and ectoderm (TUBB3) [65] markers during spontaneous EB differentiation (Additional file 1: Fig. S1G). These results indicated that *HAND* KO cell lines exhibited comparable pluripotency to the parental cell line.

We then performed cardiomyocyte differentiation with WT and *HAND* KO cell lines. After 10 days’ differentiation, we examined the expression of *HAND1* or/and *HAND2* in *H1*-KO, *H2*-KO and *H1*/*H2*-dKO cell lines-derived cardiomyocytes, which showed significantly decreased *HAND1* or/and *HAND2* expressions compared with WT cells (Fig. 1G). Moreover, HAND1 or/and HAND2 protein was not detected by western blot (Fig. 1H). These results demonstrated that we have successfully established *HAND1*/*2* single and double KO cell lines which could be used for functional research.

### *HAND1* deficiency promoted SHF and its derived cardiomyocyte differentiation

As *HAND1* was known to be expressed in the mesoderm lineage in mouse and human embryo development [21, 31], we conducted immunofluorescence analysis to investigate the impact of *HAND1* deficiency on mesoderm differentiation. At differentiation day 2, the expression of TBXT, a mesoderm marker [66], was comparable between WT and *H1*-KO cells (Additional file 1: Fig. S2A). At differentiation day 3, qPCR analysis showed the expression of cardiac mesoderm marker *MESP1* was similar between WT and *H1*-KO cells (Additional file 1: Fig. S2B). These results suggested that *HAND1* did not participate in the process of mesoderm and cardiac mesoderm induction from hESCs.

**Fig. 2:**
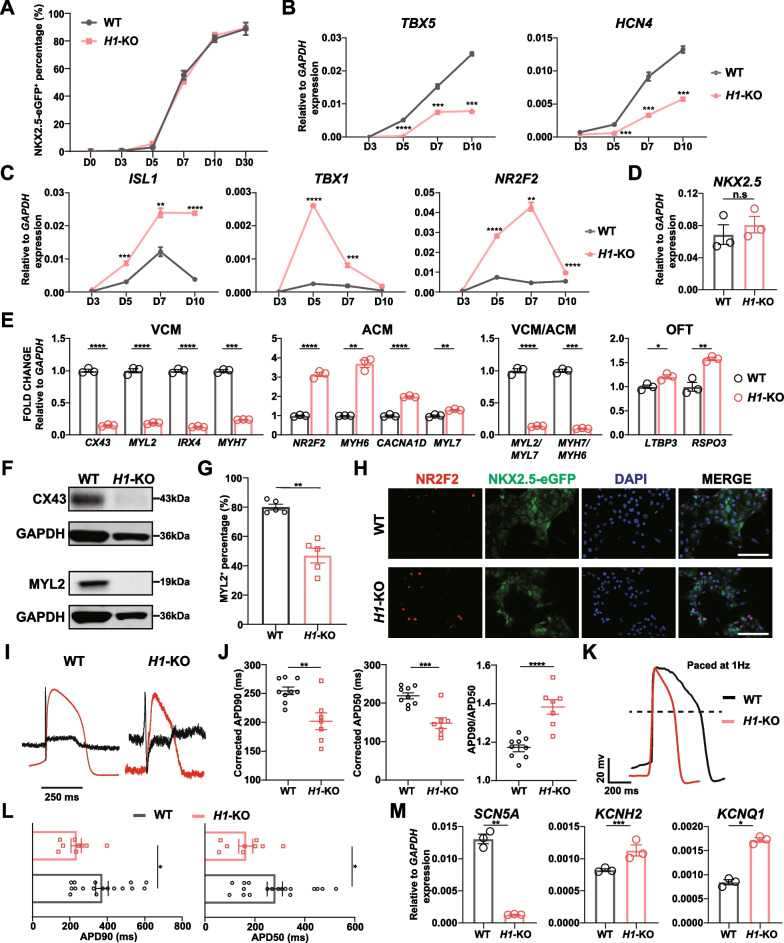
*HAND1* deficiency promoted SHF and its derived cardiomyocyte differentiation.** A** The percentage of NKX2.5-eGFP^+^ cells in WT and *H1*-KO cells at different time points of cardiomyocyte differentiation (n = 3). **B**, **C** Expression of FHF (**B**) and SHF (**C**) markers in early cardiomyocyte differentiation of WT and *H1*-KO cells. Relative to *GAPDH* expression (n = 3). Unpaired *t* test. **D**, **E** Expression of *NKX2.5* (**D**) and ventricular, atrial and outflow tract cardiomyocyte (VCM, ACM and OFT) markers (**E**) in WT and *H1*-KO-derived day 30 cardiomyocytes. Relative to *GAPDH* expression (n = 3). Unpaired *t* test. **F** Western blot analysis of CX43 and MYL2 expression in WT and *H1*-KO-derived day 30 cardiomyocytes. GAPDH served as loading control. Corresponding uncropped full-length gels and blots are presented in Additional file 8: Fig. S8. **G** The percentage of MYL2^+^ cardiomyocytes in WT and *H1*-KO cells at differentiation day 30 (n = 5). **H** Immunofluorescence staining of NR2F2 in WT and *H1*-KO-derived day 30 cardiomyocytes. Scale bar = 100 μm. **I** The field potential and simulated action potential recorded by MEA in WT and *H1*-KO-derived day 30 cardiomyocytes. Black lines represented the field potential while red lines represented the simulated action potential. **J** Comparison of corrected APD90, APD50 and APD90/APD50 ratio in WT and *H1*-KO-derived day 30 cardiomyocytes (n ≥ 7). Unpaired *t* test. **K** The action potential paced by 1Hz recorded by whole-cell patch clamp in WT and *H1*-KO-derived differentiation day 30 cardiomyocytes. **L** Comparison of APD90 and APD50 in WT and *H1*-KO-derived day 30 cardiomyocytes (n ≥ 9). Unpaired *t* test. **M** Expression of ion channels of WT and *H1*-KO-derived differentiation day 30 cardiomyocytes. Relative to *GAPDH* expression (n = 3). Unpaired *t* test. *p < 0.05, **p < 0.01, ***p < 0.001, ****p < 0.0001. n.s: non-significant

We then analyzed the cardiomyocyte differentiation of *H1*-KO cells. Similar to WT hESCs, some beating *H1*-KO cardiomyocytes were first observed at day 7. Furthermore, by flow cytometry, no difference in the percentage of NKX2.5-eGFP^+^ cells in WT and *H1*-KO cells was detected during differentiation (Fig. 2A, Additional file 1: Fig. S2C), consistent with the transcriptional level of *NKX2.5* (Additional file 1: Fig. S2D). The absence of *HAND1* did not influence the pan-cardiomyocyte differentiation efficiency.

The FHF and SHF were determined at cardiac mesoderm stage during differentiation [19, 67], and *HAND1* is a marker of FHF [18]. So, we next investigated the function of *HAND1* in cardiac lineage commitment during early cardiomyocyte differentiation. By examining the expression of heart-field specific markers from days 3 to 10 (cardiac mesoderm stage to cardiomyocyte stage), we revealed that *H1*-KO cells exhibited a notable reduction in the expression of FHF markers, *TBX5* and *HCN4* [16, 17], compared to WT cells from days 5 to 10 (Fig. 2B). Conversely, the expression of SHF marker *ISL1* was significantly upregulated from day 5 (Fig. 2C). In particular, as early as day 3, the expression of aSHF and pSHF markers, *TBX1*, *SIX1*, *FOXC2*, and *NR2F2*, *HOXA1*, *HOXB1*, *ALDH1A2*, started to display higher expression level compared with WT cells at different time points until day 7 or day 10. For example, *TBX1* expression increased from differentiation days 3 to 5, then began to decrease until differentiation day 10 while *NR2F2* was upregulated from differentiation days 3 to 7, then downregulated during differentiation days 7 to 10 (Fig. 2C, Additional file 1: Fig. S2E, F). These results demonstrated that *HAND1* deficiency promoted SHF lineage from cardiac mesoderm stage whereas impaired the FHF progenitors.

To distinguish the effects of abnormal early cardiac lineage specification, we explored the characteristics of *H1*-KO cardiomyocytes at differentiation day 30. Firstly, the *H1*-KO-derived cardiomyocytes displayed comparable levels of *NKX2.5*, same as the flow cytometry analysis of NKX2.5-eGFP^+^ cells at day 30 (Fig. 2A, D). Then, we focused on comparing the gene expression related to ventricular, atrial and OFT cardiomyocytes, which were primarily differentiated from FHF, pSHF and aSHF, respectively. The *H1*-KO-derived cardiomyocytes exhibited reduced expression of ventricular genes, including *CX43*, *MYL2*, *IRX4* and *MYH7* [67, 68], and increased atrial genes, including *NR2F2*, *MYH6*, *CACNA1D* and *MYL7* [67] (Fig. 2E). The decreased ratio of *MYL2/MYL7* and *MYH7/MYH6* [69] also reflected the reduced ventricular cardiomyocyte differentiation (Fig. 2E). Meanwhile, some OFT cardiomyocyte markers, *LTBP3* and *RSPO3* [20, 70], increased as well (Fig. 2E). Western blot analysis confirmed the reduced expression of CX43 and MYL2 at the protein level (Fig. 2F). Immunofluorescence staining also showed the expression of CX43 decreased in *H1*-KO cardiomyocytes (Additional file 1: Fig. S2G), which could affect intercellular ion movement and impulse conduction by disrupting the function of gap junctions [71]. The percentage of MYL2^+^ cardiomyocytes decreased in *H1*-KO cells (Fig. 2G). Besides, immunofluorescence staining and western blot showed that the expression of NR2F2 increased in *H1*-KO cardiomyocytes (Fig. 2H, Additional file 1: Fig. S2H). Those results reflected that *HAND1* deficiency promoted cardiomyocytes to express atrial and OFT but not ventricular cardiomyocyte markers, which was consistent with the effect of *HAND1* on early cardiac lineage differentiation.

To further characterize the *H1*-KO cardiomyocytes, we used LEAP induction to transform field potential into action potential. Figure 2I shows the relationship between field potential and LEAP induction of action potential recorded by the MEA. Compared to WT, the *H1*-KO cardiomyocytes had a shortened corrected APD90 and APD50 and increased APD90/APD50 ratio, which were similar to the electrophysiological characteristics of atrial-like cardiomyocytes (Fig. 2J). As the MEA recorded the electrophysiological characteristics of bulk cardiomyocytes, we further performed whole-cell patch clamp to compare the electrophysiological properties of single cells. We found that *H1*-KO cardiomyocytes displayed relatively short APD and comparable action potential amplitude (Fig. 2K, Additional file 1: Fig. S2I). The APD90 and APD50 of *H1*-KO-derived cardiomyocytes were shorter than WT cardiomyocytes (Fig. 2L), which aligned with the MEA findings. Additionally, to explore the molecular foundations that caused shortened APD in *H1*-KO cardiomyocytes, we checked the expression of ion channels. In *H1*-KO cardiomyocytes, the expression of *SCN5A* which encodes the subunits of sodium channel Na_v_1.5 and is essential for depolarization of cardiomyocyte [72], reduced (Fig. 2M, Additional file 1: Fig. S2J). Conversely, the expression of *KCNH2* and *KCNQ1* which mediate the repolarization current of *I*_kr_ and *I*_ks_, respectively [72], increased (Fig. 2M). Both of these changes contributed to the shortened APD [73, 74]. These results were consistent with the inference that *HAND1* deficiency decreased ventricular cardiomyocyte differentiation while promoted atrial cardiomyocyte differentiation.

### *HAND2* knockout impaired SHF-derived cardiomyocyte differentiation

**Fig. 3:**
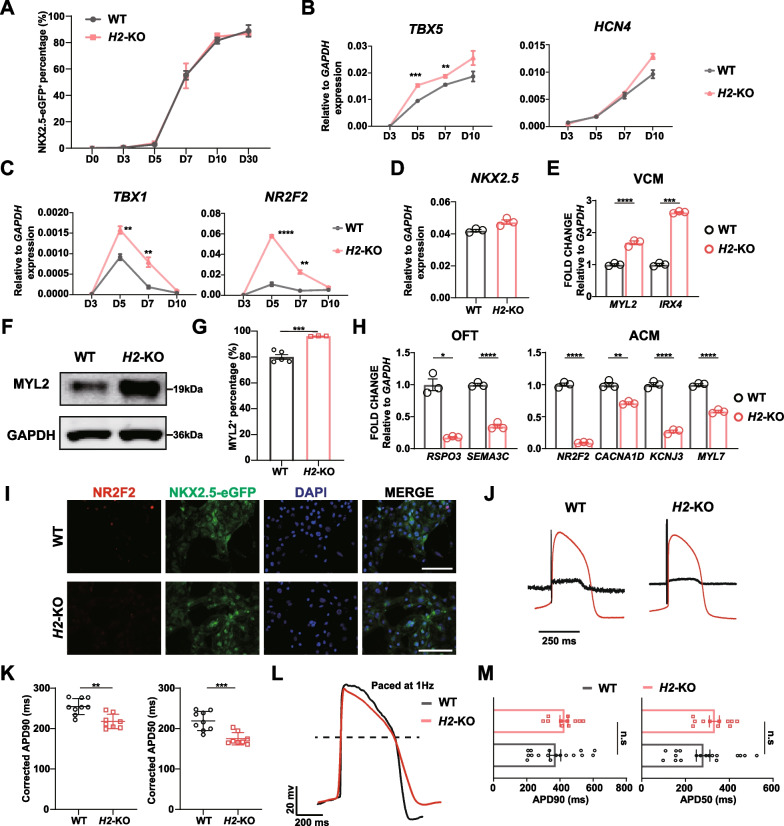
*HAND2* knockout impaired SHF-derived cardiomyocyte differentiation. **A** The percentage of NKX2.5-eGFP^+^ cells in WT and *H2*-KO cells at different time points of cardiomyocyte differentiation (n = 3). **B**, **C**. Expression of FHF (**B**) and SHF (**C**) markers in early cardiomyocyte differentiation of WT and *H2*-KO cells. Relative to *GAPDH* expression (n = 3). Unpaired *t* test. **D**, **E** Expression of *NKX2.5* (**D**) and ventricular cardiomyocyte (VCM) markers (**E**) in WT and *H2*-KO-derived differentiation day 30 cardiomyocytes. Relative to *GAPDH* expression (n = 3). Unpaired *t* test. **F** Western blot analysis of MYL2 expression in WT and *H2*-KO-derived differentiation day 30 cardiomyocytes. GAPDH served as loading control. Corresponding uncropped full-length gels and blots are presented in Additional file 8: Fig. S9. **G** The percentage of MYL2^+^ cardiomyocytes in WT and *H2*-KO cells at differentiation day 30 (n ≥ 3). **H** Expression of atrial and OFT cardiomyocyte (ACM, OFT) markers in WT and *H2*-KO-derived differentiation day 30 cardiomyocytes. Relative to *GAPDH* expression (n = 3). Unpaired *t* test. **I** Immunofluorescence staining of NR2F2 in WT and *H2*-KO-derived differentiation day 30 cardiomyocytes. Scale bar = 100 μm. **J** The field potential and simulated action potential recorded by MEA in WT and *H2*-KO-derived differentiation day 30 cardiomyocytes. Black lines represented the field potential while red lines represented the simulated action potential. **K** Comparison of corrected APD90 and APD50 in WT and *H2*-KO-derived differentiation day 30 cardiomyocytes (n ≥ 8). Unpaired *t* test. **L** The action potential paced by 1Hz recorded by whole-cell patch clamp in WT and *H2*-KO-derived day 30 cardiomyocytes. **M** Comparison of APD90 and APD50 in WT and *H2*-KO-derived differentiation day 30 cardiomyocytes (n ≥ 11). Unpaired *t* test. *p < 0.05, **p < 0.01, ***p < 0.001, ****p < 0.0001. n.s: non-significant

As *HAND2* was also expressed in mesoderm, but later than *HAND1* [21, 31], we examined TBXT and *MESP1* expression at days 2 and 3, respectively, which showed no difference between WT and *H2*-KO cells (Additional file 1: Fig. S3A, B). We then differentiated *H2*-KO hESCs into cardiomyocytes, which exhibited comparable differentiation kinetics to WT cells (Fig. 3A, Additional file 1: Fig. S3C) with cell beating initiated at day 7. Additionally, the expression of *NKX2.5* in *H2*-KO cells was comparable to WT cells during differentiation days 3 to 10 (Additional file 1: Fig. S3D). Thus, *HAND2* deficiency had no significant effects on pan-cardiomyocyte differentiation efficiency.

We next detected the expression of FHF and SHF markers during early cardiac lineage specification in *H2*-KO cells. qPCR analysis revealed that *HAND2* knockout resulted in higher expression of *TBX5* and comparable expression of *HCN4* compared to WT cells during differentiation days 5 to 7 (Fig. 3B). Meanwhile, the expression of *ISL1* increased at differentiation day 5 and the expression of aSHF and pSHF markers was also globally higher than that of WT cells and reduced to the same level as WT cells at differentiation day 10 (Fig. 3C, Additional file 1: Fig. S3E). These results indicated that *HAND2* differed from *HAND1* in their roles in early cardiac lineage commitment.

We next explored the characteristics of *H2*-KO cardiomyocytes. At differentiation day 30, *H2*-KO cardiomyocytes showed comparable expression of *NKX2.5* (Fig. 3D). Unlike *H1*-KO cardiomyocytes, *HAND2* knockout did not interrupt the ventricular cardiomyocyte differentiation as indicated by upregulated expression of *MYL2* and *IRX4* (Fig. 3E). Additionally, the expression of MYL2 protein and the percentage of MYL2^+^ cardiomyocytes in *H2*-KO cells increased (Fig. 3F, G), while the expression of CX43 in WT and *H2*-KO cardiomyocytes was similar (Additional file 1: Fig. S3F). Single-cell analysis of *Hand2*-null mouse heart revealed that *Hand2* did not affect the specification of the right ventricular cardiomyocytes, but it did impact the formation of OFT cardiomyocytes [30]. We therefore determined the expression of OFT cardiomyocyte markers, *RSPO3* and *SEMA3C* [19], which reduced at day 30 (Fig. 3H). The *H2*-KO cardiomyocytes also exhibited reduced expression of atrial cardiomyocyte markers, including *NR2F2*, *CACNA1D*, *KCNJ3* and *MYL7* (Fig. 3H), indicating that *HAND2* affected the formation of SHF-derived cardiomyocytes. Immunofluorescence staining of NR2F2 showed very few *H2*-KO cardiomyocytes were NR2F2 positive (Fig. 3I), which was further confirmed by western blot (Additional file 1: Fig. S3G).

*HAND2* deficiency cells showed elevated expression of aSHF and pSHF markers. However, the expression of SHF-derived atrial and OFT cardiomyocyte markers decreased. We speculated that the differentiation of SHF progenitors into atrial and OFT cardiomyocytes might be impaired in the absence of *HAND2*. We therefore analyzed the expression of progenitor markers in *H2*-KO-derived differentiation day 30 cardiomyocytes. Interestingly, the expression of cardiac progenitor cell markers, *PDGFRA*, *FLK1* and *ISL1*, which expressed in SHF progenitors [75, 76], was still at high level in *H2*-KO differentiated cells, indicating the impeded SHF differentiation in *H2*-KO cells (Additional file 1: Fig. S3H).

Finally, we used MEA to characterize the electrophysiological properties of differentiation day 30 *H2*-KO cardiomyocytes, which displayed comparable action potential pattern to WT cardiomyocytes (Fig. 3J). However, the corrected APD90 and APD50 were found to be slightly shorter compared to WT cardiomyocytes (Fig. 3K). Additionally, whole-cell patch clamp recorded action potentials with more concentrated distribution of APD90 and APD50 compared to WT cardiomyocytes (Fig. 3L, M), although with no significant difference, indicating that *H2*-KO cardiomyocytes had a tendency toward ventricular cardiomyocytes, consistent with their gene expression profile (Fig. 3E–G). In addition, the action potential amplitude of *H2*-KO cardiomyocytes was comparable to WT cardiomyocytes as well (Additional file 1: Fig. S3I). Next, we detected the expression of ion channels in *H2*-KO cardiomyocytes. The expression of *SCN5A* was moderately decreased in *H2*-KO cardiomyocytes (Additional file 1: Fig. S3J). Meanwhile, the expression of *KCNH2* and *KCNQ1* was similar between these two groups (Additional file 1: Fig. S3J), validating the electrophysiological characters of *H2*-KO cardiomyocytes.

### *HAND1*/*2* double knockout impeded the differentiation of cardiomyocytes and impaired their electrophysiological activity

It was generally accepted that *HAND1* and *HAND2* were partially redundant in heart development [27]. We therefore examined the mRNA and protein expression of HAND1 in *H2*-KO and HAND2 in *H1*-KO cell lines, which revealed that HAND2 was significantly upregulated in *H1*-KO cells during early cardiomyocyte differentiation whereas HAND1 was only slightly upregulated in *H2*-KO cells before differentiation day 7 (Additional file 1: Fig. S4A-C). These results indicated that *HAND1*/*2* could have some complementary function with *HAND1* playing more important roles in our cardiomyocyte differentiation system. To clarify the function of *HAND1*/*2*, we generated the *H1*/*H2*-dKO cell lines for further analysis.

**Fig. 4:**
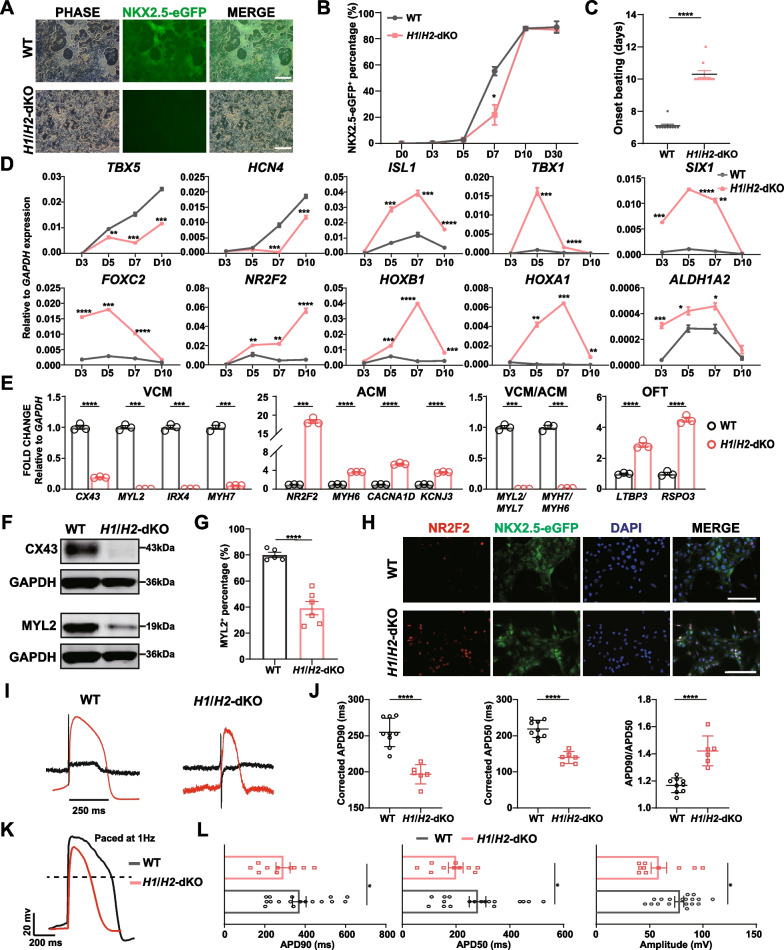
*HAND1*/*2* double knockout impeded the differentiation of cardiomyocytes and impaired their electrophysiological activity. **A** The phase and fluorescence images of WT and *H1*/*H2*-dKO cells at differentiation day 7. **B** The percentage of NKX2.5-eGFP^+^ cells in WT and *H1*/*H2*-dKO cells at different time points of cardiomyocyte differentiation (n = 3). Unpaired *t* test. **C** The beating onset of WT and *H1*/*H2*-dKO-derived cardiomyocytes (n = 10). Unpaired *t* test. **D** Expression of FHF and SHF markers in early cardiomyocyte differentiation of WT and *H1*/*H2*-dKO cells. Relative to *GAPDH* expression (n = 3). Unpaired *t* test. **E** Expression of ventricular, atrial and outflow tract cardiomyocyte (VCM, ACM and OFT) markers in WT and *H1*/*H2*-dKO-derived day 30 cardiomyocytes. Relative to *GAPDH* expression (n = 3). Unpaired *t* test. **F** Western blot analysis of CX43 and MYL2 expression in day 30 WT and *H1*/*H2*-dKO cardiomyocytes. Corresponding uncropped full-length gels and blots are presented in Additional file 8: Fig. S10. **G** The percentage of MYL2.^+^ cardiomyocytes in WT and *H1*/*H2*-dKO cells at differentiation day 30 (n = 3). **H** Immunofluorescence staining of NR2F2 in WT and *H1*/*H2*-dKO-derived day 30 cardiomyocytes. Scale bar = 100 μm. **I** The field potential and simulated action potential recorded by MEA in WT and *H1*/*H2*-dKO-derived day 30 cardiomyocytes. Black lines represented the field potential while red lines represented the action potential. **J** Comparison of corrected APD90, APD50 and APD90/APD50 ratio in WT and *H1*/*H2*-dKO-derived differentiation day 30 cardiomyocytes (n ≥ 6). Unpaired *t* test. **K** The action potential paced by 1Hz recorded by whole-cell patch clamp in WT and *H1*/*H2*-dKO-derived differentiation day 30 cardiomyocytes. **L** Comparison of APD90, APD50 and action potential amplitude in WT and *H1*/*H2*-dKO-derived day 30 cardiomyocytes (n ≥ 10). Unpaired *t* test. *p < 0.05, **p < 0.01, ***p < 0.001, ****p < 0.0001

Firstly, immunofluorescence staining of TBXT at differentiation day 2 revealed comparable fluorescence intensity in *H1*/*H2*-dKO cells, suggesting mesoderm differentiation was not impaired (Additional file 1: Fig. S4D). Also, no difference in *MESP1* expression was observed at differentiation day 3 (Additional file 1: Fig. S4E). We then applied the same method to induce the differentiation of *H1*/*H2*-dKO hESCs into cardiomyocytes. Of note, at day 7, the NKX2.5-eGFP fluorescence was much weaker in *H1*/*H2*-dKO cells compared to WT cells (Fig. 4A), consistent with lower percentage of NKX2.5-eGFP^+^ cells in *H1*/*H2*-dKO determined by flow cytometry (Fig. 4B). However, at day 10, the *H1*/*H2*-dKO NKX2.5-eGFP^+^ population reached to comparable level as that of WT cells (Fig. 4B, Additional file 1: Fig. S4F). Additionally, *H1*/*H2*-dKO cardiomyocytes exhibited delayed beating onset which was not observed until differentiation days 10–12 (Fig. 4C). The expression of *NKX2.5* in *H1*/*H2*-dKO cells also lagged behind WT cells (Additional file 1: Fig. S4G). The delayed cardiomyocyte differentiation of *H1*/*H2*-dKO hESCs indicated that *HAND1* and *HAND2* had overlapping functions in the early stage of cardiomyocyte differentiation.

We next determined the expression of FHF and SHF markers in early cardiomyocyte differentiation of *H1*/*H2*-dKO cells. Specifically, the expression of FHF markers, *TBX5* and *HCN4*, decreased as that of *H1*-KO cells, while the expression of SHF markers, including *ISL1*, *TBX1*, *SIX1*, *FOXC2*, *NR2F2*, *HOXB1*, *HOXA1* and *ALDH1A2*, increased with similar trend as that of *H1*-KO cells, except that the expression of *NR2F2* kept in an upward trend from differentiation days 7 to 10 and was significantly higher than that in WT and *H1*-KO cells at differentiation day 10 (Figs. 2B, C, 4D, Additional file 1: Fig. S2E, F). Besides, the expression of *TBX1* and *SIX1* was much higher than that of *H1*-KO cells (Figs. 2C, 4D, Additional file 1: Fig. S2E). These results indicated that *H1*/*H2*-dKO hESCs biased toward SHF differentiation.

To assess the effects of *H1*/*H2*-dKO on cardiomyocyte subtype differentiation, we profiled differentiation day 30 cells. The *H1*/*H2*-dKO cardiomyocytes exhibited comparable level of *NKX2.5* (Additional file 1: Fig. S4H). The ventricular cardiomyocyte markers *CX43*, *MYL2*, *IRX4* and *MYH7* were downregulated in *H1*/*H2*-dKO cardiomyocytes, while both atrial cardiomyocyte markers, *NR2F2*, *MYH6*, *CACNA1D* and *KCNJ3*, and OFT cardiomyocyte markers, *LTBP3* and *RSPO3*, were upregulated (Fig. 4E). The ratio of *MYL2*/*MYL7* and *MYH7*/*MYH6* also decreased in *H1*/*H2*-dKO cardiomyocytes, resembling the trend observed in *H1*-KO cardiomyocytes (Figs. 2E, 4E). We also found that the upregulated expression of atrial cardiomyocyte markers *NR2F2* and *CACNA1D* and the OFT cardiomyocyte markers *LTBP3* and *RSPO3* was significantly higher in *H1*/*H2*-dKO cells compared with *H1*-KO cells (Figs. 2E, 4E), suggesting *H1*/*H2*-dKO cells were more prone to SHF-derived cardiomyocyte differentiation than *H1*-KO cells. Furthermore, western blot analysis confirmed the reduced expression of CX43 and MYL2 (Fig. 4F). Concomitantly, the percentage of MYL2^+^ cardiomyocytes in *H1*/*H2*-dKO cells also decreased (Fig. 4G). Immunofluorescence staining revealed decreased expression of CX43 in *H1*/*H2*-dKO cardiomyocytes as well (Additional file 1: Fig. S4I), whereas the expression of NR2F2 significantly increased (Fig. 4H, Additional file 1: Fig. S4J). These results were consistent with the more SHF progenitors generated during early differentiation of *H1*/*H2*-dKO hESCs.

To further characterize the *H1*/*H2*-dKO cardiomyocyte subtypes, we conducted MEA and whole-cell patch clamp to analyze the electrophysiological properties of these cells. MEA revealed that *H1*/*H2*-dKO cardiomyocytes exhibited shortened APD, including corrected APD90 and APD50 (Fig. 4I, J). The ratio of APD90/APD50 increased in *H1*/*H2*-dKO cardiomyocytes compared to WT cardiomyocytes (Fig. 4J). Whole-cell patch clamp confirmed that *H1*/*H2*-dKO cardiomyocytes displayed shortened APD (Fig. 4K, L), indicating that these cells exhibited electrophysiological characteristics more akin to atrial-like cardiomyocytes. Besides, the amplitude of *H1*/*H2*-dKO cardiomyocytes’ action potential was reduced compared to WT cardiomyocytes (Fig. 4L), which was not observed in *H1*-KO and *H2*-KO cardiomyocytes (Additional file 1: Figs. S2I, S3I), suggesting greater impairment in conduction speed and electrical activity of cardiomyocytes [77]. The reduced expression of *SCN5A* and increased expression of *KCNH2* and *KCNQ1* (Additional file 1: Fig. S4K, L), which were similar to *H1*-KO cardiomyocytes (Fig. 2M, Additional file 1: Fig. S2J), partially accounted for the changes in electrophysiological characteristics of *H1*/*H2*-dKO cardiomyocytes. Overall, these findings demonstrated that *H1*/*H2*-dKO hESCs were prone to SHF-derived cardiomyocyte differentiation with severely impaired electrophysiological activity.

### Transcriptomic characterization of *H1*-KO, *H2*-KO and *H1*/*H2*-dKO cardiomyocytes

**Fig. 5:**
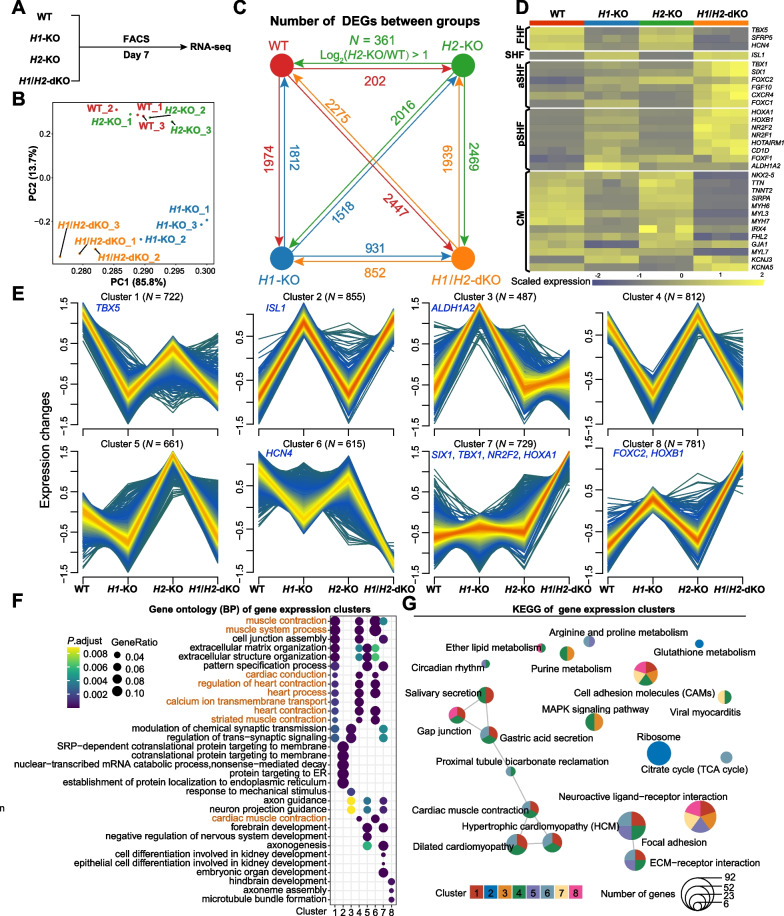
Identifying the transcriptomic characteristics of *H1*-KO*, H2*-KO and *H1/H2*-dKO cardiomyocytes. **A** Schematic diagram of the samples for RNA-seq. WT, *H1*-KO, *H2*-KO and *H1*/*H2*-dKO cardiomyocytes were collected at differentiation day 7. **B** PCA analysis of WT, *H1*-KO, *H2*-KO and *H1*/*H2*-dKO cardiomyocytes. Each group had three duplicates. **C** The DEGs between different groups. DEGs were defined with |fold change (FC)|> 2 and adjusted *P*-value < 0.05. **D** The expression of representative FHF, SHF and cardiomyocyte (CM) genes in RNA-seq. **E** Gene expression cluster analysis of RNA-seq. Genes included in clustering were at least differentially expressed in one comparison in (**C**). **F** GO enrichment analysis of each gene expression cluster. **G** KEGG pathway enrichment analysis of each gene expression cluster

To provide an integrated view of the implications of HAND1- and HAND2-dependent transcriptional changes, we performed RNA-seq of WT, *H1*-KO, *H2*-KO and *H1*/*H2*-dKO cardiomyocytes sorted at differentiation day 7 (a time point that NKX2.5-eGFP^+^ cells emerged and the majority of the differentiating cardiomyocytes diverged) (Figs. 5A, 4A, B, Additional file 1: Fig. S4G). The mapping statistics and PCA analysis showed the high quality of RNA-seq data, with an average of about 25 million mapped reads (Additional file 3: Table S3) and a high correlation between duplicates (Fig. 5B). PCA analysis showed WT and *H2*-KO cells were transcriptionally similar, while *H1*-KO and *H1*/*H2*-dKO cells were distinct from WT and *H2*-KO cells. Further pairwise differential expression analysis identified 3786 DEGs (1812 upregulated and 1974 downregulated) between WT and *H1*-KO cells; 563 DEGs (361 upregulated and 202 downregulated) between WT and *H2*-KO cells; and 4722 DEGs (2275 upregulated and 2447 downregulated) between WT and *H1*/*H2*-dKO cells (Fig. 5C), indicating a significant shift in gene expression profile in *H1*-KO and *H1*/*H2*-dKO cells. Notably, *HAND2* expression was elevated in *H1*-KO cells while *HAND1* expression did not change significantly in *H2*-KO cells (Additional file 1: Fig. S5), similar to the qPCR and western blot data at differentiation day 7 (Additional file 1: Fig. S4A-C). We also observed the downregulated expression of FHF markers (such as *TBX5* and *HCN4*) and upregulated expression of SHF markers (such as *ISL1*, *TBX1*, *SIX1*, *FOXC2*, *NR2F2*, *HOXA1*, *HOXB1* and *ALDH1A2*) in *H1*-KO and *H1*/*H2*-dKO cells (Fig. 5D), consistent with our experimental results (Figs. 2B, C, 4D and Additional file 1: Fig. S2E, F). These findings suggested that *HAND1* played a more crucial role in FHF development than *HAND2*.

To figure out the dynamic gene expression changes between WT, *H1*-KO, *H2*-KO and *H1*/*H2*-dKO cardiomyocytes, we utilized the fuzzy c-means algorithm to cluster gene expression profiles into different groups. Eight distinct clusters were identified (Fig. 5E, Additional file 4: Table S4), representing different expression kinetics in response to *H1*-KO, *H2*-KO and *H1*/*H2*-dKO. Among them, the changes of the above-mentioned markers were consistent with Fig. 5D, indicating the reliability of the clustering. Clusters 1, 4, 5 and 6 shared similar trends with genes downregulated, and clusters 2, 3, 7 and 8 represented the upregulated genes in *H1*-KO and *H1*/*H2*-dKO cells (Fig. 5E). Furthermore, we applied GO and KEGG enrichments to predict the underlying functions of each gene cluster (Fig. 5F, G). As expected, clusters 1, 4 and 6 (clusters with genes downregulated in *H1*-KO, *H2*-KO and *H1*/*H2*-dKO cells) were highly enriched in similar GO and KEGG terms associated with cardiomyocyte contraction, development, and heart diseases (hypertrophic cardiomyopathy and dilated cardiomyopathy) (Fig. 5F, G). The other clusters (clusters 2, 3, 5, 7 and 8) were enriched in various cluster-specific terms, such as protein targeting and location (Cluster 2), other organ developments (clusters 5, 7 and 8) (Fig. 5F, G, Additional file 5, 6: Table S5, S6). Taken together, *H1*-KO, *H2*-KO and *H1*/*H2*-dKO cardiomyocytes manifested corresponding transcriptome changes in accordance with the *H1*-KO, *H2*-KO and *H1*/*H2*-dKO cardiomyocyte differentiation phenotypes.

### *HAND1*/*2* modulated cardiomyocyte differentiation through TBX5

**Fig. 6:**
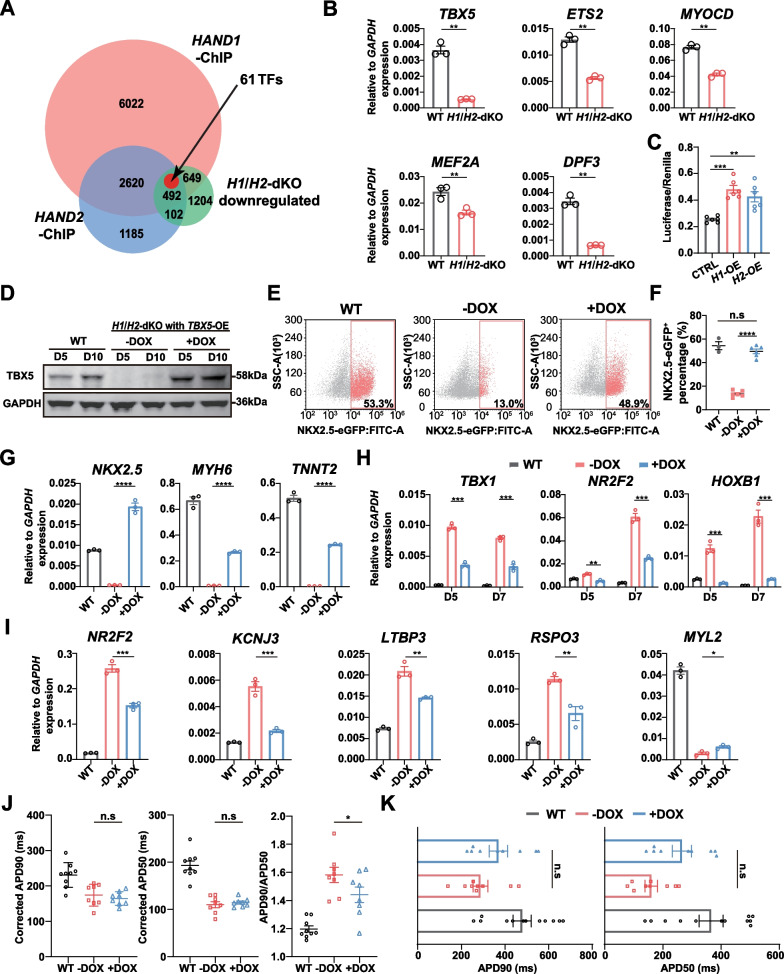
*HAND1*/*2* modulated cardiomyocyte differentiation through *TBX5*. **A** Venn diagram of HAND1 and HAND2 ChIP-seq and downregulated DEGs in *H1*/*H2*-dKO cells compared to WT cells in RNA-seq. **B** Expression of some TFs in WT and *H1*/*H2*-dKO sorted NKX2.5-eGFP^+^ cells at differentiation day 7. Relative to *GAPDH* expression (n = 3). Unpaired *t* test. **C** The ratio of Luciferase and Renilla activity in HAND1 and HAND2 overexpression 293T cells (n = 6). CTRL represented 293T transfected with pGL3-*TBX5*-*Luciferase* and *Renilla* only. One-way ANOVA. **D** TBX5 overexpression after DOX induction verified by western blot at differentiation days 5 and 10. GAPDH served as loading control. Corresponding uncropped full-length gels and blots are presented in Additional file 8: Fig. S11. **E**, **F** The percentage of NKX2.5-eGFP^+^ cells in WT and *TBX5*-OE cells (−DOX and + DOX) at differentiation day 7 (n ≥ 3). One-way ANOVA. **G** Expression of *NKX2.5*, *MYH6* and *TNNT2* in WT and *TBX5*-OE cells (−DOX and + DOX) at differentiation day 7. Relative to *GAPDH* expression (n = 3). One-way ANOVA. **H** Expression of SHF markers in WT and *TBX5*-OE cells (−DOX and + DOX) at differentiation days 5 and 7. Relative to *GAPDH* expression (n = 3). One-way ANOVA. **I** Expression of atrial, OFT and ventricular cardiomyocyte markers in WT and *TBX5*-OE cells (−DOX and + DOX)-derived differentiation day 30 cardiomyocytes. Relative to *GAPDH* expression (n = 3). One-way ANOVA. **J** Comparison of corrected APD90, APD50 and APD90/APD50 ratio recorded by MEA in WT and *TBX5*-OE cells (−DOX and + DOX)-derived differentiation day 30 cardiomyocytes (n ≥ 8). One-way ANOVA. **K** Comparison of APD90 and APD50 recorded by whole-cell patch clamp in WT and *TBX5*-OE cells (−DOX and + DOX)-derived day 30 cardiomyocytes (n ≥ 9). One-way ANOVA. *p < 0.05, **p < 0.01, ***p < 0.001, ****p < 0.0001. n.s: non-significant

To elucidate the underlying mechanism of *HAND1*/*2* in cardiac lineage differentiation, we downloaded the chromatin immunoprecipitation sequencing (ChIP-seq) database of HAND1 and HAND2 (GSM1505812 and GSM1505811), which was conducted at the mesoderm stage during differentiation from hESCs, equivalent to days 2 to 3 in our differentiation protocol [78]. Comprehensive analysis of RNA-seq and ChIP-seq data unveiled that among the genes downregulated in *H1*/*H2*-dKO cardiomyocytes, 649 and 102 genes were regulated by HAND1 and HAND2 alone, respectively. Meanwhile, 492 genes were co-regulated by HAND1 and HAND2, which included 61 TFs activated by HAND1/2 (Fig. 6A, Additional file 1: Table S2). Among these TFs, several were known to play important roles in heart development, such as TBX5 [79], MEF2 family [80, 81], MYOCD [82], ETS2 [83, 84] and NKX2.5 [85, 86] (Fig. 6A, Additional file 1: Table S2). This suggested that the absence of *HAND1* and *HAND2* could lead to dysregulation of cardiomyocyte differentiation gene network. The reduced expression of these genes in the *H1*/*H2*-dKO NKX2.5-eGFP^+^ cardiomyocytes at differentiation day 7 was validated by qPCR (Fig. 6B). We then focused on *TBX5* which is a FHF marker and was significantly downregulated in *H1*/*H2*-dKO NKX2.5-eGFP^+^ cardiomyocytes (Fig. 6B). Previous studies showed that *TBX5*-deficient hPSCs exhibited decreased cardiomyocyte differentiation efficiency and delayed onset of beating, along with delayed *NKX2.5* expression [79]. In addition, single-cell sequencing of heterozygous and homozygous *TBX5* deletion hPSC-derived cardiomyocytes revealed a higher proportion of atrial-like cardiomyocytes [79]. Those phenotypes were similar to *H1*/*H2*-dKO cells.

To confirm the regulation of *TBX5* by HAND1/2, we constructed a Luciferase plasmid under the control of *TBX5* promoter (Additional file 1: Fig. S6A) and co-transfected with the *HAND1* or *HAND2* overexpression plasmid (*H1*-OE, *H2*-OE) into HEK 293T cells, a commonly used cell line because of its high transfection efficiency. Both HAND1 and HAND2 activated Luciferase expression, indicating HAND1/2 regulated *TBX5* promoter activity (Fig. 6C). Subsequently, we constructed *TBX5* overexpression *H1*/*H2*-dKO cell line with DOX-inducible Tet-On system (referred as *TBX5*-OE). In the presence of DOX (+ DOX), the expression of *TBX5* was activated. We confirmed the completely restored TBX5 protein expression in + DOX group at differentiation days 5 and 10 (Fig. 6D).

With DOX induction, at differentiation day 7, we observed that *TBX5* overexpression restored the NKX2.5-eGFP^+^ population to the level as that of WT cells as evidenced by fluorescence microscope (Additional file 1: Fig. S6B, C) and flow cytometry analysis (Fig. 6E, F). Moreover, the expression of cardiomyocyte markers *NKX2.5*, *MYH6* and *TNNT2* in + DOX group was also upregulated compared to −DOX group (Fig. 6G). Thus, with *TBX5* overexpression, the delayed cardiomyocyte differentiation was rescued. Intriguingly, TBX5 overexpression did not recover the beating onset of *H1*/*H2*-dKO cells as early as WT cells (Additional file 1: Fig. S6D). Next, we explored the effects of *TBX5* on *H1*/*H2*-dKO cardiac lineage commitment. We observed significant reduction in some SHF markers, including *TBX1*, *SIX1*, *NR2F2*, *HOXA1*, *HOXB1* and *ALDH1A2* from differentiation days 5 to 7 and *FOXC2* at differentiation day 7 in + DOX group, whereas *HCN4* slightly increased at differentiation day 7 (Fig. 6H, Additional file 1: Fig. S6E). At differentiation day 30, TBX5 overexpressing cardiomyocytes exhibited reduced expression of SHF-derived atrial and OFT cardiomyocyte markers, including *NR2F2*, *KCNJ3* and *LTBP3*, *RSPO3* (Fig. 6I), while the ventricular cardiomyocyte marker *MYL2* were only slightly upregulated (Fig. 6I).

Finally, we performed MEA and whole-cell patch clamp to analyze the electrophysiology characteristics of *H1*/*H2*-dKO cardiomyocytes with and without TBX5 overexpression. Corrected APD90 and APD50 of TBX5 overexpressing cardiomyocytes recorded by MEA were not statistically different from the −DOX group, whereas the ratio of APD90/APD50 decreased in TBX5 overexpressing cardiomyocytes (Fig. 6J). The results of whole-cell patch clamp showed increase of APD90 and APD50 in + DOX cardiomyocytes compared with −DOX group, although the difference was not statistically significant (Fig. 6K). These results indicated that the delayed cardiomyocyte differentiation in *H1*/*H2*-dKO cells was caused by reduced *TBX5* expression in the absence of *HAND1*/*2*. However, the tendency of differentiation into SHF-derived cardiomyocytes was only partially rescued, suggesting that the function of *HAND1*/*2* in cardiac lineage commitment was partially dependent on TBX5.

## Discussion

In this study, we investigated the function of *HAND1*/*2* in human cardiac lineage commitment and differentiation by inducing differentiation of *HAND* depleted *NKX2.5*^*eGFP*^ H9 hESCs into cardiomyocytes. We revealed that *HAND1* deficiency, as well as *HAND1/2* double knockout hESCs, biased to differentiate into SHF lineage and its derived cardiomyocytes at the expense of FHF progenitors, while *HAND2* knockout impaired cardiomyocyte differentiation from SHF progenitors. Moreover, *HAND1*/*2* double knockout also delayed the onset of cardiomyocyte differentiation. These results highlighted the difference and redundance of *HAND1*/*2* during the differentiation from cardiac mesoderm to cardiomyocytes.

*HAND1* deficiency led to obviously upregulated expression of SHF markers from cardiac mesoderm stage and maintained at higher level than WT throughout early cardiomyocyte differentiation. Conversely, *HAND1* knockout downregulated the expression of FHF markers from the cardiac progenitor stage. It was known that *HAND1* was initially expressed in mesoderm [19, 22, 31], but its function in human early cardiac lineage commitment was not completely clear. We verified that *HAND1* was one of the key factors that determine the FHF and SHF fates from cardiac mesoderm. And with the differentiation of cardiac progenitor cells, *HAND1*-deficient cardiac mesoderm cells eventually produced more SHF-derived cardiomyocytes instead of FHF-derived cardiomyocytes. Meanwhile, the elevated *HAND2* in *H1*-KO cells could also contribute to the generation of SHF-derived cardiomyocytes, as *HAND2* promoted SHF development [29].

In *H2*-KO cells, the expression of SHF markers slightly increased from cardiac mesoderm stage, while the expression of atrial and OFT cardiomyocyte markers downregulated and the cardiac progenitor markers maintained at relatively high level at differentiation day 30, indicating that *HAND2* deficiency impaired SHF differentiation. These results were consistent with the findings in *Hand2*-null mice which had increased aSHF and pSHF progenitors and impaired OFT differentiation [30]. The elevated MYL2^+^ cardiomyocyte population and the ventricular-like action potentials of these cardiomyocytes further corroborated the properties of *H2*-KO cardiomyocytes. Notably, we found that *HAND1* was upregulated in *H2*-KO cells, which was in line with a previous report that *Hand1* was robustly expressed in ventricular and lateral mesoderm in *Hand2* deficiency embryos [25]. Thus, *HAND1* in *H2*-KO cells could contribute to FHF-derived ventricular cardiomyocytes and restrict SHF differentiation. Considering that *HAND2* was primarily expressed in human atrial cardiomyocytes [35] and upregulated in atrial cardiomyocyte differentiation [37], we speculated that *HAND2* deficiency could also impair the SHF-derived atrial cardiomyocytes. Unfortunately, the cardiomyocytes generated in our differentiation system were mainly ventricular subtype [67, 87]. The role of *HAND2* in atrial cardiomyocyte differentiation from hPSCs warrants further investigation.

During cardiomyocyte differentiation, distinct from *H2*-KO cells, both *H1*-KO and *H1*/*H2*-dKO cells displayed highly expressed SHF markers and later produced cardiomyocytes with atrial-like action potentials, indicating that *H1*-KO cells were in proximity to *H1*/*H2*-dKO cells, which was confirmed by transcriptome analysis. At the transcriptional level, *H1*/*H2*-dKO cells were much closer to *H1*-KO rather than *H2*-KO cells, and the SHF markers were upregulated in both *H1*-KO and *H1*/*H2*-dKO cells, although higher in *H1*/*H2*-dKO cells. The severer impairment of cardiomyocyte differentiation in *H1/H2*-dKO cells could be explained by that *HAND1* determined the fate of FHF and SHF at cardiac mesoderm stage. On the basis that *HAND1* knockout altered the fate of cardiac progenitors, *HAND2* deficiency led to further impairment of subsequent cardiomyocyte differentiation and electrophysiological activity, as the case in *Hand1*/*2* deficiency mice which manifested severe heart defects and embryonic death [27]. Intriguingly, the cardiomyocyte differentiation process was delayed in *H1/H2*-dKO cells, suggesting that to safeguard the timing of cardiac differentiation, either *HAND1* or *HAND2* was required.

According to the ChIP-seq and RNA-seq analyses, we validated that TBX5 was a key downstream TF activated by HAND1/2. *TBX5* deficiency in hPSCs led to delayed cardiac differentiation and biased toward atrial-like cardiomyocytes [79], similar to the phenotypes observed in *HAND1*/*2* defects. Overexpressing *TBX5* in *H1*/*H2*-dKO cells was able to restore the cardiomyocyte differentiation efficiency to the level of WT cells at differentiation day 7. The onset beating time of *H1*/*H2*-dKO cells was not rescued by TBX5, indicating that TBX5 may not be the key regulator for the impaired contractile activity in *H1*/*H2*-dKO cardiomyocytes. Although the expression of SHF markers, as well as its derived cardiomyocyte markers, and the ratio of APD90/APD50 decreased with TBX5 overexpression, they did not recover to the level of WT cells. Therefore, for the tendency toward SHF differentiation in *H1*/*H2*-dKO cells, it was only partially rescued by TBX5 overexpression which could be attributed to the complex regulatory mechanisms involving *HAND1*/*2* during cardiomyocyte differentiation. Moreover, since the expression of *TBX5* was detected from differentiation day 5, later than *HAND1* (Figs. 1B, 2B), we hypothesized that the overexpression of TBX5 could not reverse the already determined SHF fate at cardiac mesoderm stage (day 3) due to *HAND1* deficiency.

Both HAND1 and HAND2 were capable of activating the *TBX5* promoter. However, we found that *TBX5* expression was decreased in *H1*-KO and *H1*/*H2*-dKO cells, and slightly increased in *H2*-KO cells. We speculated that *HAND1* exerted stronger regulation over *TBX5*, and in *H1-*KO cells, though the elevated expression of *HAND2* could not restore *TBX5* expression, it could compensate for *HAND1* depletion by interacting with *TBX5* [88], as evidenced by the onset of cardiomyocyte differentiation which was normal in *H1*-KO cells, but delayed in *H1/H2*-dKO cells. In *H2*-KO cells, the upregulated *HAND1* compensated for the loss of *HAND2* to activate *TBX5* expression. In addition, TBX5 overexpression rescued the delayed cardiomyocyte differentiation in *H1/H2*-dKO cells, also verifying *TBX5* as a downstream target of HAND1/2. Consequently, to timely initiate cardiomyocyte differentiation, either *HAND1*, *HAND2* or sufficient level of *TBX5* was required.

The study has several limitations. Firstly, it should be acknowledged that there are vast differences between in vitro and in vivo conditions for cardiomyocyte differentiation. The complex in vivo microenvironment where the human embryonic heart develops is regulated by fine-tuned transcription factors and signaling pathways [1, 89], making it challenging to fully replicate this niche in vitro. Therefore, the findings of the study may be restricted to some extent. For further validation and mechanistic investigation, 3D cardiac organoid models or in vivo experiments on large animals are necessary. Secondly, we found numerous important cardiac developmental TFs such as DPF3, MEF2A and ETS2, which may be modulated by HAND1/2 during cardiac differentiation. In this study, we primarily focused on TBX5, which partially rescued the defects of *H1*/*H2*-dKO cells in cardiomyocyte differentiation. Therefore, the extensive mechanisms by which *HAND1*/*2* regulate the cardiac lineage commitment await being elucidated in future.

## Conclusion

We revealed the specific and redundant function of *HAND1*/*2* in human heart development by providing comprehensive survey of *HAND1*/*2* on cardiac lineage commitment and differentiation from pluripotent stem cells. Furthermore, *TBX5* was verified as one of the key factors in *HAND1*/*2* gene regulatory network during cardiac organogenesis. These findings may potentially facilitate new treatment strategies for CHDs.

### Supplementary Information

**Additional file 1: Fig. S1**. Establishment of *H1*-KO, *H2-*KO and *H1*/*H2*-dKO hESC lines. **Fig. S2**. *HAND1* deficiency promoted SHF and its derived cardiomyocyte differentiation. **Fig. S3**. *HAND2* knockout impaired SHF-derived cardiomyocyte differentiation. **Fig. S4**. *HAND1*/*2* double knockout impeded the differentiation of cardiomyocytes and impaired their electrophysiological activity. **Fig. S5**. Expression of *HAND1* in *H2*-KO cells and *HAND2* in *H1*-KO cells in sorted cardiomyocytes at differentiation day 7 by RNA-seq. **Fig. S6**. *HAND1*/*2* modulated cardiomyocyte differentiation through TBX5. **Table S1**. Primers used in PCR and qPCR in the experiments. **Table S2**. Transcription factors regulated by HAND1 and HAND2 in *H1*/*H2*-dKO downregulated genes.

**Additional file 2.** The video of beating WT cardiomyocytes at differentiation day 7.

**Additional file 3:** **Table S3**. The number of reads mapped per sample of RNA-seq data.

**Additional file 4.** **Table S4**. Genes list of each gene expression cluster in Fig. 5E.

**Additional file 5.** **Table S5**. GO enrichment analysis of each gene expression cluster in Fig. 5F.

**Additional file 6.** **Table S6**. KEGG pathway enrichment analysis of each gene expression cluster in Fig. 5G.

**Additional file 7.** **Table S7**. Expression profiling of the sorted WT and *HAND* KO cardiomyocytes at differentiation day 7 by RNA-seq and the comparison between samples. The transcriptional levels were shown as TPM.

**Additional file 8.** **Fig. S7**. Full-length blots of Fig. 1H. Western blot analysis of HAND1 and HAND2 expression in KO cell lines-derived day 10 cardiomyocytes. **Fig. S8**. Full-length blots of Fig. 2F and Fig. S2H, J. Western blot analysis of CX43, MYL2, NR2F2 and SCN5A expression in *H1*-KO-derived day 30 cardiomyocytes. **Fig. S9**. Full-length blots of Fig. 3F and Fig. S3G. Western blot analysis of MYL2 and NR2F2 expression in *H2*-KO-derived day 30 cardiomyocytes. **Fig. S10**. Full-length blots of Fig. 4F and Fig. S4B, C, J, L. Western blot analysis of CX43, MYL2, NR2F2 and SCN5A expression in *H1*/*H2*-dKO-derived day 30 cardiomyocytes. Western blot analysis of HAND1 and HAND2 expression in *H2*-KO and *H1*-KO cells, respectively. **Fig. S11**. Full-length blots of Fig. 6D. Western blot analysis of TBX5 expression in TBX5-OE cell lines (-DOX/+DOX) at differentiation days 5 and 10.

## Data Availability

The ChIP-seq of HAND1/2 datasets used and analyzed during the current study are available from the GEO/NCBI (GSM1505812 and GSM1505811, https://www.ncbi.nlm.nih.gov/geo/query/acc.cgi?acc=GSE61475). The raw RNA-seq data of sorted NKX2.5-eGFP^+^ cells at differentiation day 7 have been deposited into the CNGB Sequence Archive (CNSA) of the China National GeneBank DataBase (CNGBdb) with accession number CNP0005227 (https://db.cngb.org/search/project/CNP0005227/).

## Abbreviations

ACM: Atrial cardiomyocyte
APD: Action potential duration
aSHF: Anterior second heart field
CHD: Congenital heart disease
CM: Cardiomyocyte
FHF: First heart field
hESCs: Human embryonic stem cells
hPSCs: Human pluripotent stem cells
LEAP: Local extracellular action potential
MEA: Microelectrode array
OFT: Outflow tract
pSHF: Posterior second heart field
SHF: Second heart field
TF: Transcription factor
VCM: Ventricular cardiomyocyte
