# Supplementary material for: HAND factors regulate cardiac lineage commitment and differentiation from human pluripotent stem cells
Source: Stem Cell Res Ther. 2024 Feb 5;15:31. doi: 10.1186/s13287-024-03649-9 (PMC10845658; doi:10.1186/s13287-024-03649-9)
Supplement: Supplementary file 1 — Additional file 1: Fig. S1. Establishment of H1-KO, H2-KO and H1/H2-dKO hESC lines. Fig. S2. HAND1 deficiency promoted SHF and its derived cardiomyocyte differentiation. Fig. S3. HAND2 knockout impaired SHF-derived cardiomyocyte differentiation. Fig. S4. HAND1/2 double knockout impeded the differentiation of cardiomyocytes and impaired their electrophysiological activity. Fig. S5. Expression of HAND1 in H2-KO cells and HAND2 in H1-KO cells in sorted cardiomyocytes at differentiation day 7 by RNA-seq. Fig. S6. HAND1/2 modulated cardiomyocyte differentiation through TBX5. Table S1. Primers used in PCR and qPCR in the experiments. Table S2. Transcription factors regulated by HAND1 and HAND2 in H1/H2-dKO downregulated genes. [file 13287_2024_3649_MOESM1_ESM.docx]

**Additional file 1**

**Fig. S1**


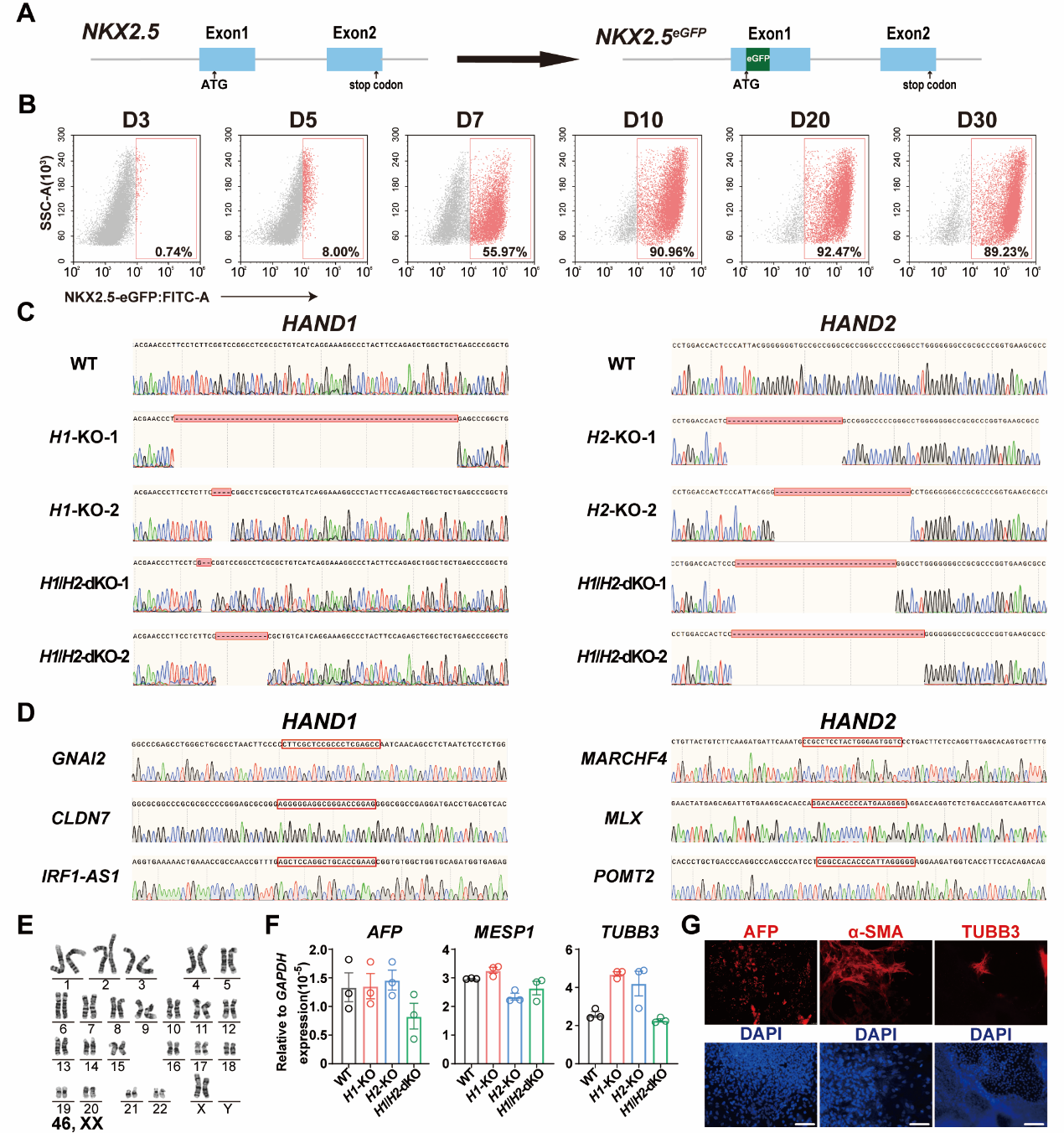


**Fig. S1** Establishment of *H1*-KO, *H2*-KO and *H1*/*H2*-dKO hESC lines. **A** Schematic diagram of the construction of *NKX2.5^eGFP^* H9 cell line. **B** Flow cytometry analysis of NKX2.5-eGFP^+^ cells at differentiation day 3, day 5, day 7, day 10, day 20 and day 30. **C** Sanger sequencing of the genomic PCR products of *HAND1* or/and *HAND2* in *H1*-KO, *H2*-KO and *H1*/*H2*-dKO hESC lines. **D** Representative Sanger sequencing of genomic PCR products of predicted off-target loci for *HAND1* and *HAND2* sgRNA in *H1*/*H2*-dKO hESC lines. **E** Karyotyping analysis of *H1*/*H2*-dKO hESCs. **F** Expression of *AFP*, *MESP1* and *TUBB3* in WT and *HAND* KO hESC lines. Relative to *GAPDH* expression (n = 3). **G** Immunofluorescence staining of endoderm (AFP), mesoderm (α-SMA) and ectoderm (TUBB3) in EB differentiation of *H1*/*H2*-dKO cells. Scale bar = 100 μm.

**Fig. S2**


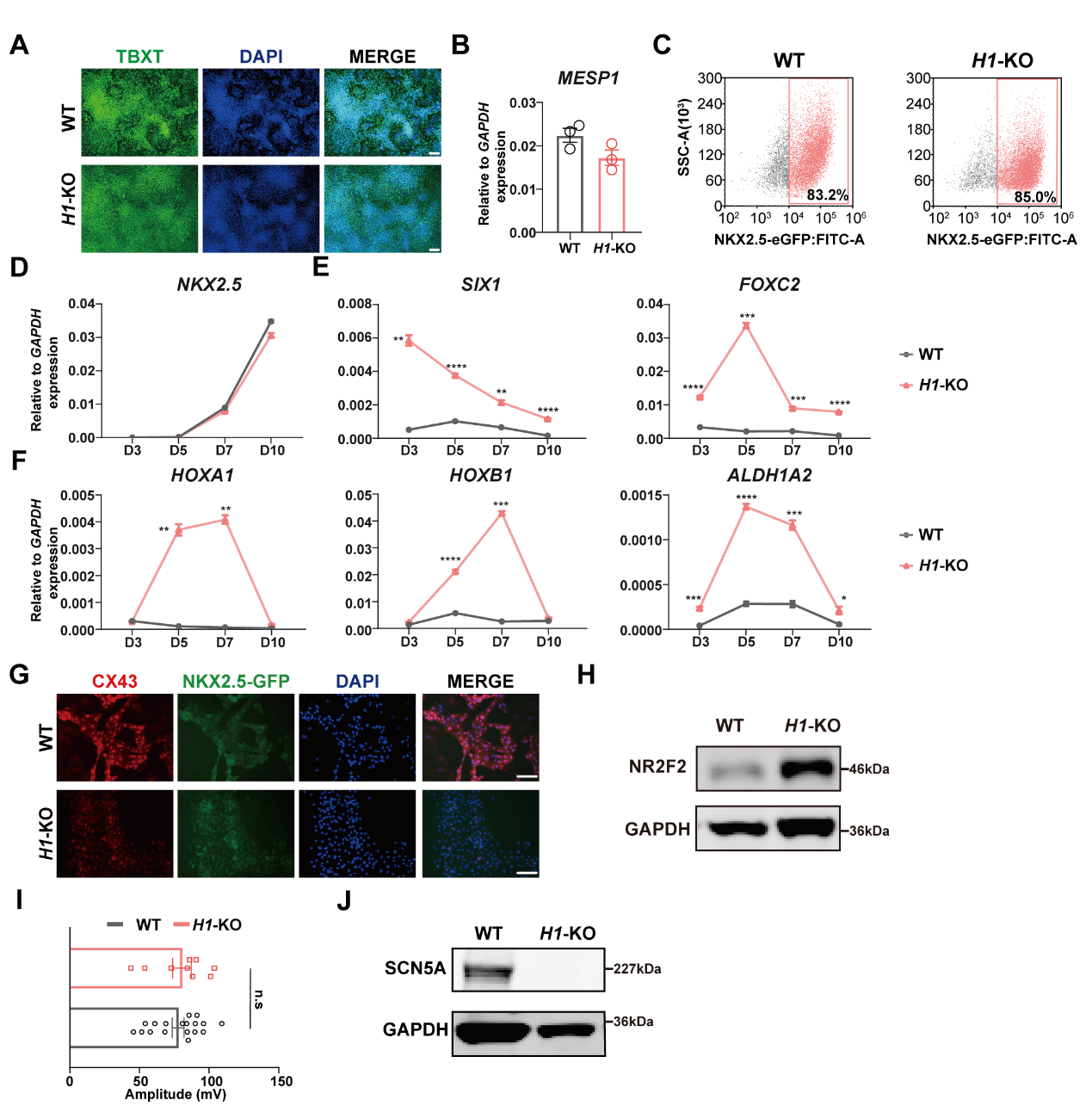


**Fig. S2** *HAND1* deficiency promoted SHF and its derived cardiomyocyte differentiation. **A** Immunofluorescence staining of TBXT in WT and *H1*-KO cells at differentiation day 2. Scale bar = 100 μm. **B** Expression of *MESP1* in WT and *H1*-KO cells at differentiation day 3. Relative to *GAPDH* expression (n = 3). **C** The percentage of NKX2.5-eGFP^+^ of WT and *H1*-KO cells at differentiation day 10. **D** Expression of *NKX2.5* in early cardiomyocyte differentiation of WT and *H1*-KO cells. Relative to *GAPDH* expression (n = 3). **E, F** Expression of SHF markers in early cardiomyocyte differentiation of WT and *H1*-KO cells. Relative to *GAPDH* expression (n = 3). Unpaired *t*-test. **G** Immunofluorescence staining of CX43 in WT and *H1*-KO-derived differentiation day 30 cardiomyocytes. Scale bar = 100 μm. **H** Western blot analysis of NR2F2 expression in WT and *H1*-KO-derived differentiation day 30 cardiomyocytes. GAPDH served as loading control. Corresponding uncropped full-length gels and blots are presented in Additional file 8: Fig. S8. **I** Comparison of action potential amplitude in WT and *H1*-KO-derived differentiation day 30 cardiomyocytes (n ≥ 9). Unpaired *t*-test. **J** Western blot analysis of SCN5A expression in WT and *H1*-KO-derived differentiation day 30 cardiomyocytes. GAPDH served as loading control. Corresponding uncropped full-length gels and blots are presented in Additional file 8: Fig. S8. *p < 0.05, **p < 0.01, ***p < 0.001, ****p < 0.0001. n.s: non-significant.

**Fig. S3**


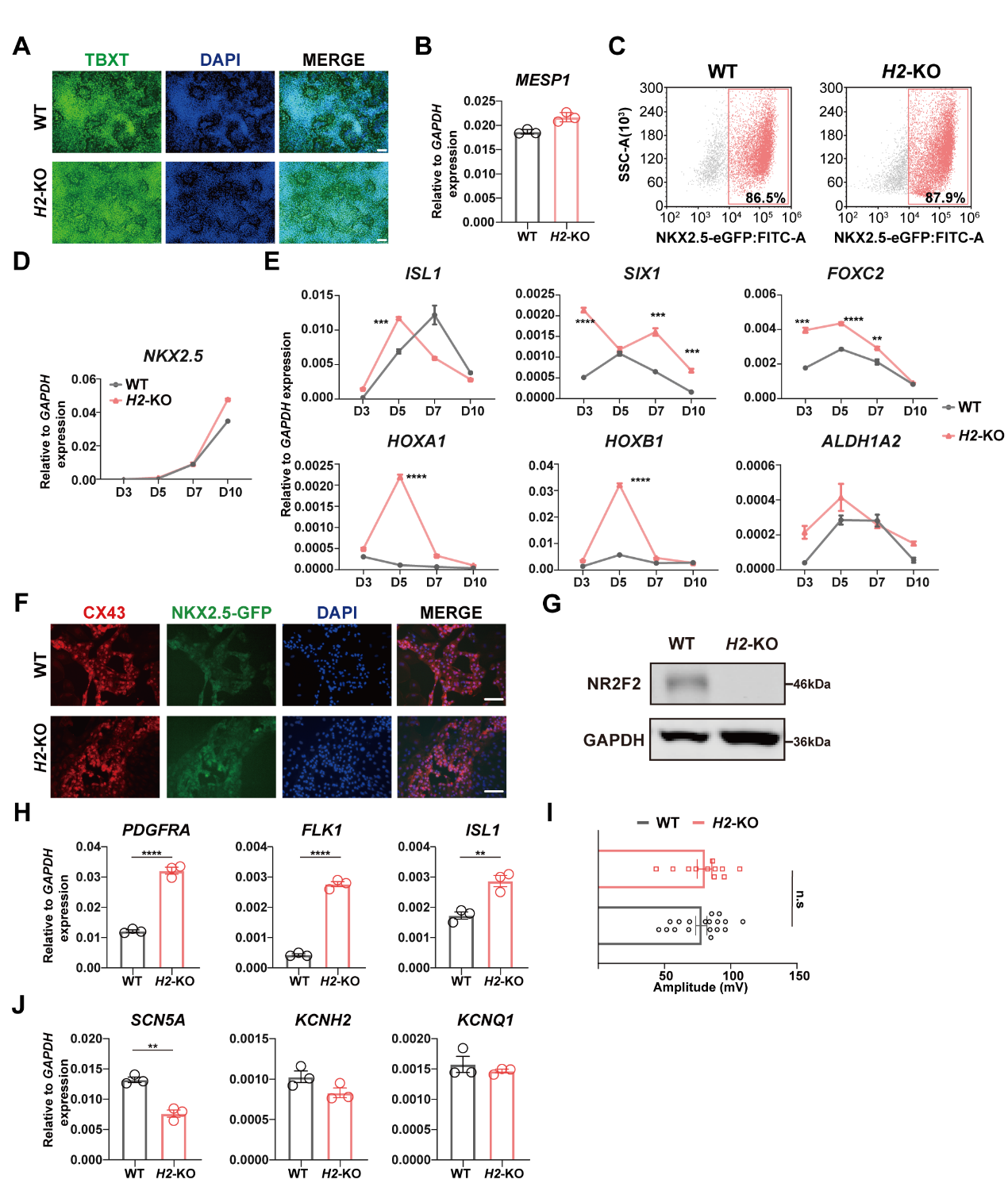


**Fig. S3** *HAND2* knockout impaired SHF derived cardiomyocyte differentiation. **A** Immunofluorescence staining of TBXT in WT and *H2*-KO cells at differentiation day 2. Scale bar = 100 μm. **B** Expression of *MESP1* in WT and *H2*-KO cells at differentiation day 3. Relative to *GAPDH* expression (n = 3). **C** The percentage of NKX2.5-eGFP^+^ of WT and *H2*-KO cells at differentiation day 10. **D, E** Expression of *NKX2.5* (**D**) and SHF markers (**E**) in early cardiomyocyte differentiation of WT and *H2*-KO cells. Relative to *GAPDH* expression (n = 3). Unpaired *t*-test. **F** Immunofluorescence staining of CX43 in WT and *H2*-KO-derived differentiation day 30 cardiomyocytes. Scale bar = 100 μm. **G** Western blot analysis of NR2F2 expression in WT and *H2*-KO-derived differentiation day 30 cardiomyocytes. GAPDH served as loading control. Corresponding uncropped full-length gels and blots are presented in Additional file 8: Fig. S9. **H** Expression of cardiac progenitor cell markers in WT and *H2*-KO-derived differentiation day 30 cardiomyocytes. Relative to *GAPDH* expression (n = 3). Unpaired *t*-test. **I** Comparison of action potential amplitude in WT and *H2*-KO-derived differentiation day 30 cardiomyocytes (n ≥ 11). Unpaired *t*-test. **J** Expression of ion channels in WT and *H2*-KO-derived day 30 cardiomyocytes. Relative to *GAPDH* expression (n = 3). Unpaired *t*-test. **p < 0.01, ***p < 0.001, ****p < 0.0001. n.s: non-significant.

**Fig. S4**


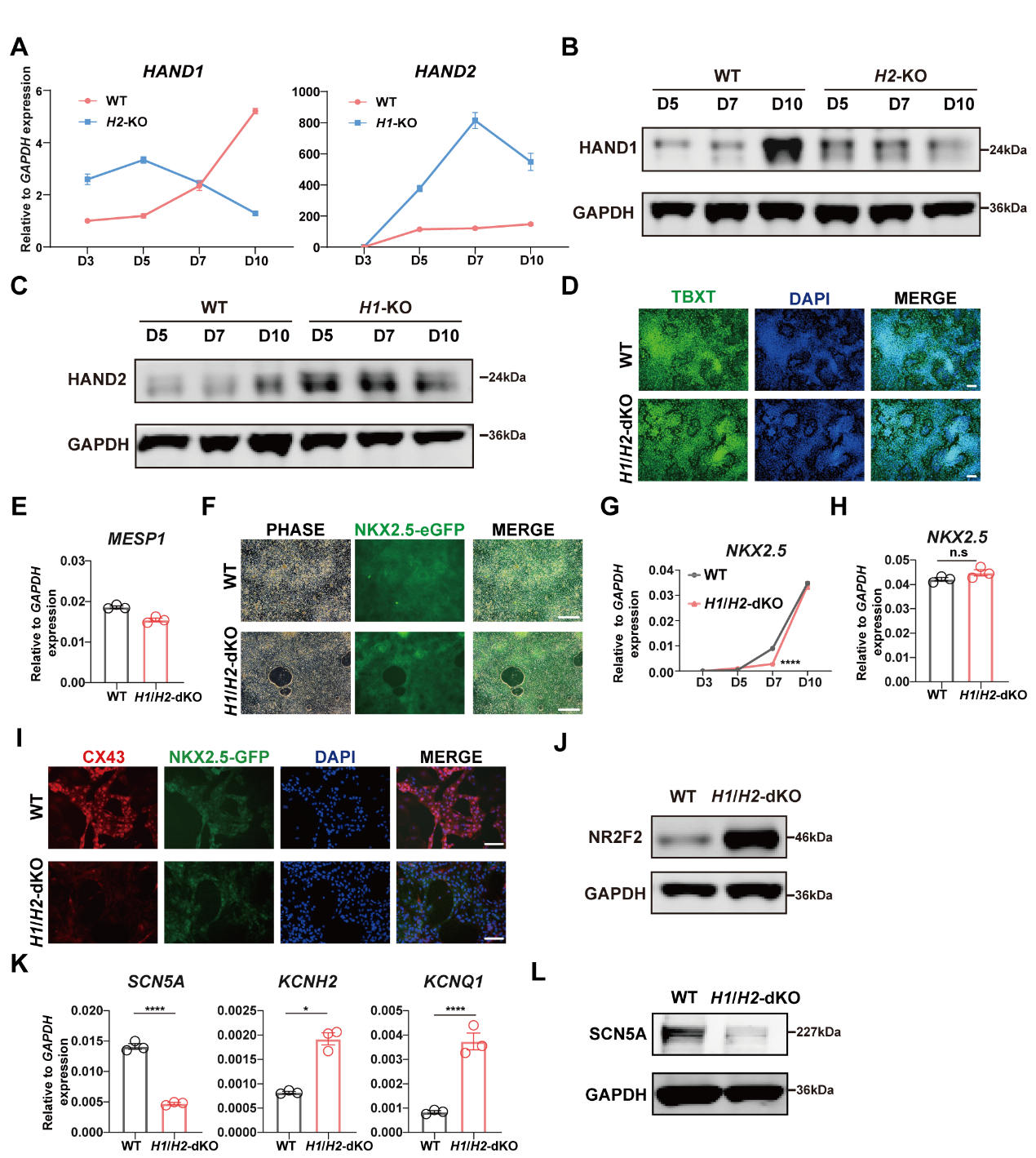


**Fig. S4** *HAND1*/*2* double knockout impeded the differentiation of cardiomyocytes and impaired their electrophysiological activity. **A-C** Expression of *HAND1* in *H2*-KO cells (**A**, **B**) and *HAND2* in *H1*-KO cells (**A**, **C**) during early cardiomyocyte differentiation. Relative to *GAPDH* expression (n = 3) for **A**. GAPDH served as loading control for **B** and **C**. Corresponding uncropped full-length gels and blots are presented in Additional file 8: Fig. S10. **D** Immunofluorescence staining of TBXT in WT and *H1*/*H2*-dKO cells at differentiation day 2. Scale bar = 100 μm. **E** Expression of *MESP1* in WT and *H1*/*H2*-dKO cells at differentiation day 3. Relative to *GAPDH* expression (n = 3). **F** The phase and fluorescence images of WT and *H1*/*H2*-dKO cells at differentiation day 10. Scale bar = 100 μm. **G** Expression of *NKX2.5* in early cardiomyocyte differentiation of WT and *H1*/*H2*-dKO cells. Relative to *GAPDH* expression (n = 3). Unpaired *t*-test. **H** Expression of *NKX2.5* in WT and *H1*/*H2*-dKO-derived differentiation day 30 cardiomyocytes. Relative to *GAPDH* expression (n = 3). Unpaired *t*-test. **I** Immunofluorescence staining of CX43 in WT and *H1*/*H2*-dKO-derived differentiation day 30 cardiomyocytes. Scale bar = 100 μm. **J** Western blot analysis of NR2F2 expression in WT and *H1*/*H2*-dKO-derived differentiation day 30 cardiomyocytes. GAPDH served as loading control. Corresponding uncropped full-length gels and blots are presented in Additional file 8: Fig. S10. **K** Expression of ion channels in WT and *H1*/*H2*-dKO-derived differentiation day 30 cardiomyocytes. Relative to *GAPDH* expression (n = 3). Unpaired *t*-test. **L** Western blot of SCN5A in WT and *H1*/*H2*-dKO-derived differentiation day 30 cardiomyocytes. GAPDH served as loading control. Corresponding uncropped full-length gels and blots are presented in Additional file 8: Fig. S10. *p < 0.05, ****p < 0.0001. n.s: non-significant.

**Fig. S5**


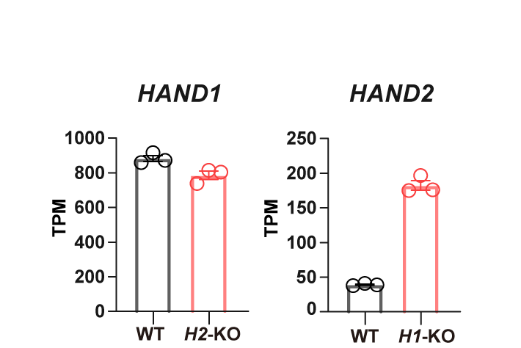


**Fig. S5** Expression of *HAND1* in *H2*-KO cells and *HAND2* in *H1*-KO cells in sorted cardiomyocytes at differentiation day 7 by RNA-seq. The transcriptional levels were shown as transcripts per million (TPM).

**Fig. S6**


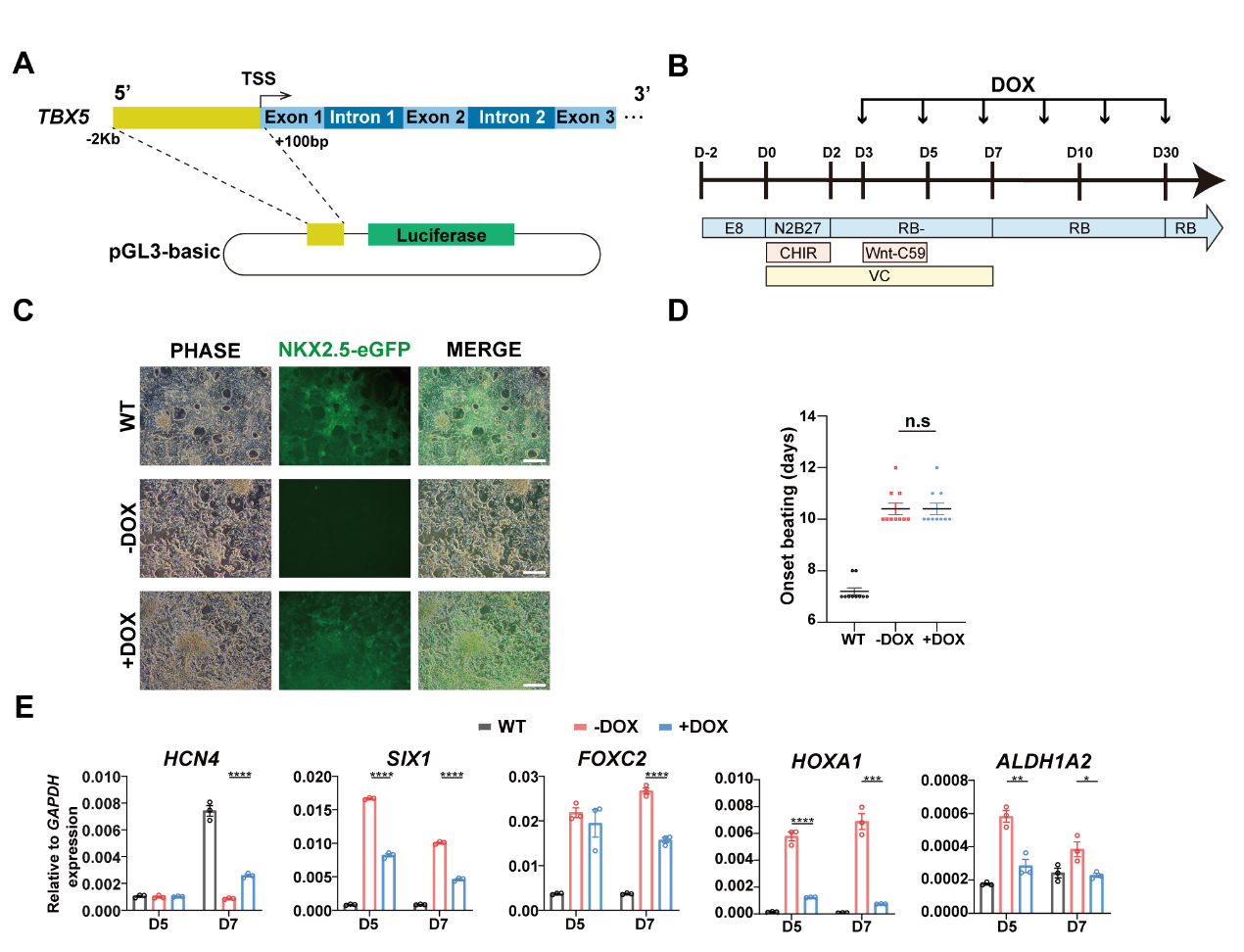


**Fig. S6** *HAND1*/*2* modulated cardiomyocyte differentiation through *TBX5*. **A** Schematic diagram of the construction of pGL3-*TBX5*-*Luciferase* vector. **B** Schematic diagram of the induction of TBX5 expression with DOX during cardiomyocyte differentiation. **C** The phase and fluorescence images of WT and *TBX5*-OE cells (-DOX and +DOX) at differentiation day 7. Scale bar = 100 μm. **D** The beating onset of WT and *TBX5*-OE cells (-DOX and +DOX) derived cardiomyocytes (n = 10). Unpaired t-test. **E** Expression of *HCN4* and SHF markers in WT and *TBX5*-OE cells (-DOX and +DOX) at differentiation days 5 and 7. Relative to *GAPDH* expression (n=3). One-way ANOVA. *p < 0.05, **p < 0.01, ***p < 0.001, ****p < 0.0001.

**Table S1 Primers used in PCR and qPCR in the experiments.**

| **Primers used for vector construction** | |
| --- | --- |
| *HAND1*-OE-F | 5’CATCATTTTGGCAAAGAATTCGCCACCATGAACCTCGTGGGCAGCT3’ |
| *HAND1*-OE-R | 5’CGCGGCCGCGATTACACGCGTTCACTGGTTTAACTCCAGCGC3’ |
| *HAND2*-OE-F | 5’CATCATTTTGGCAAAGAATTCGCCACCATGAGTCTGGTAGGTGGTTTTCCC3’ |
| *HAND2*-OE-R | 5’CGCGGCCGCGATTACACGCGTTCACTGCTTGAGCTCCAGGG3’ |
| *TBX5*-OE-F | 5’GATCGGCCGGATATCGAATTCGCCACCATGGCCGACGCAGACGAG3’ |
| *TBX5*-OE-R | 5’CGCGGCCGCGATTACACGCGTTTAGCTATTGTCGCTCCACTCTGG3’ |
| *TBX5*-promoter-F | 5’AGGTACCGAGCTCTTACGCGTATAATAACAGCCAGTATTCT3’ |
| *TBX5*-promoter-R | 5’ATCGCAGATCTCGAGCCCGGGGAGGGAACTCTTCACGAAGG3’ |
| **Primers used for genotyping** | |
| *HAND1*-F | 5’GCAGTCTCTGGGGCGCATG3’ |
| *HAND1*-R | 5’ATGACGAATCTGCCTCCCT3’ |
| *HAND2*-F | 5’GGCGAAATGAGTCTGGTAGGT3’ |
| *HAND2*-R | 5’AGATGCCATTTCTCAGCCCAA3’ |
| **Primers used for qPCR** | |
| h-*GAPDH*-qF | 5’GTCTCCTCTGACTTCAACAGCG3’ |
| h-*GAPDH*-qR | 5’ACCACCCTGTTGCTGTAGCCAA3’ |
| h-*HAND1*-qF | 5’TCCAACATGAACCTCGTGGG3’ |
| h-*HAND1*-qR | 5’AGTAGGGCCTTTCCTGATGA3’ |
| h-*HAND2*-qF | 5’GGCCAAGGACGACCAGAATG3’ |
| h-*HAND2*-qR | 5’TGCTTTTCAAGATTTCGTTCAGC3’ |
| h-*OCT4*-qF | 5’CCTGAAGCAGAAGAGGATCACC3’ |
| h-*OCT4*-qR | 5’AAAGCGGCAGATGGTCGTTTGG3’ |
| h-*SOX2*-qF | 5’GCTACAGCATGATGCAGGACCA3’ |
| h-*SOX2*-qR | 5’TCTGCGAGCTGGTCATGGAGTT3’ |
| h-*NANOG*-qF | 5’CTCCAACATCCTGAACCTCAGC3’ |
| h-*NANOG*-qR | 5’CGTCACACCATTGCTATTCTTCG3’ |
| h-*AFP*-qF | 5’GCAGAGGAGATGTGCTGGATTG3’ |
| h-*AFP*-qR | 5’CGTGGTCAGTTTGCAGCATTCTG3’ |
| h-*TUBB3*-qF | 5’CAGGTACAGGTCCACGCC3’ |
| h-*TUBB3*-qR | 5’TCACTGATGACTTCCCAGAACTGT3’ |
| h-*MESP1*-qF | 5’AGCTGCACCCGAGCCGCGC3’ |
| h-*MESP1*-qR | 5’ATCCAGGTCTCCAACAGAGCCA3’ |
| h-*NKX2.5*-qF | 5’TTTGCATTCACTCCTGCGGAGACCTA3’ |
| h- *NKX2.5*-qR | 5’ACTCATTGCACGCTGCATAATCGC3’ |
| h-*TBX5*-qF | 5’TTGCATGTATGCCAGCTCTG3’ |
| h-*TBX5*-qR | 5’CTGGTAGGGTAGCCTGTCC3’ |
| h-*HCN4*-qF | 5’TCTACTCGCTGAGCGTGGACAA3’ |
| h-*HCN4*-qR | 5’GAGTTGAGGTCGTGCTGGACTT3’ |
| h-*ISL1*-qF | 5’GCAGAGTGACATAGATCAGCCTG3’ |
| h-*ISL1*-qR | 5’GCCTCAATAGGACTGGCTACCA3’ |
| h-*TBX1*-qF | 5’CGCAGTGGATGAAGCAAATCGTG3’ |
| h-*TBX1*-qR | 5’TTTGCGTGGGTCCACATAGACC3’ |
| h-*SIX1*-qF | 5’AGGTCAGCAACTGGTTTAAGAACC3’ |
| h-*SIX1*-qR | 5’GAGGAGAGAGTTGGTTCTGCTTG3’ |
| h-*FOXC2*-qF | 5’TCACCTTGAACGGCATCTACCAG3’ |
| h-*FOXC2*-qR | 5’TGACGAAGCACTCGTTGAGCGA3’ |
| h-*NR2F2*-qF | 5’TGCACGTTGACTCAGCCGAGTA3’ |
| h-*NR2F2*-qR | 5’AAGCACACTGAGACTTTTCCTGC3’ |
| h-*HOXA1*-qF | 5’CAGCGCAGACTTTTGACTGGATG3’ |
| h-*HOXA1*-qR | 5’TCCTTCTCCAGTTCCGTGAGCT3’ |
| h-*HOXB1*-qF | 5’TGCCCTTCAGAACCTAACACCC3’ |
| h-*HOXB1*-qR | 5’AGCTGCCTTGTGGTGAAGTTGG3’ |
| h-*ALDH1A2*-qF | 5’GAGTAACTCTGGAACTTGGAGGC3’ |
| h-*ALDH1A2*-qR | 5’ATGGACTCCTCCACGAAGATGC3’ |
| h-*IRX4*-qF | 5’TTGGACTCCTGGGAACATGGACAA3’ |
| h-*IRX4*-qR | 5’ATGCTTCAGGGTATCTGGCCTCTT3’ |
| h-*CX43*-qF | 5’GGAGATGAGCAGTCTGCCTTTC3’ |
| h-*CX43*-qR | 5’TGAGCCAGGTACAAGAGTGTGG3’ |
| h-*MYL2*-qF | 5’TACGTTCGGGAAATGCTGAC3’ |
| h-*MYL2*-qR | 5’TTCTCCGTGGGTGATGATG3’ |
| h-*MYL7*-qF | 5’CCGTCTTCCTCACGCTCTT3’ |
| h-*MYL7*-qR | 5’TGAACTCATCCTTGTTCACCAC3’ |
| h-*MYH7*-qF | 5’GGAGTTCACACGCCTCAAAGAG3’ |
| h-*MYH7*-qR | 5’TCCTCAGCATCTGCCAGGTTGT3’ |
| h-*MYH6*-qF | 5’TCTCCGACAACGCCTATCAGTAC3’ |
| h-*MYH6*-qR | 5’GTCACCTATGGCTGCAATGCT3’ |
| h-*KCNJ3*-qF | 5’GATCTCCATGAGGGACGGAAAAC3’ |
| h-*KCNJ3*-qR | 5’GAAGGAACTCACCCTCAGGTGT3’ |
| h-*CACNA1D*-qF | 5’CTTCGACAACGTCCTCTCTGCT3’ |
| h-*CACNA1D*-qR | 5’GCCGATGTTCTCTCCATTCGAG3’ |
| h-*SEMA3C*-qF | 5’ACCCACTGACTCAATGCAGAGG3’ |
| h-*SEMA3C*-qR | 5’CAGCCACTTGATAGATGCCTGC3’ |
| h-*LTBP3*-qF | 5’CGGTCACTACAAGTGCAACTGC3’ |
| h-*LTBP3*-qR | 5’CTTGTTCTCGCATTTGCCATCCG3’ |
| h-*RSPO3*-qF | 5’CCAGAAGGGTTGGAAGCCAACA3’ |
| h-*RSPO3*-qR | 5’CCTTCTTCGTGCATGGACTCCA3’ |
| h-*SCN5A*-qF | 5’CACGCGTTCACTTTCCTTC3’ |
| h-*SCN5A*-qR | 5’AAGAGCCGACAAATTGCCTA3’ |
| h-*KCNQ1*-qF | 5’ATGGTGCGCATCAAGGAG3’ |
| h-*KCNQ1*-qR | 5’GATGAACAGTGAGGGCTTCC3’ |
| h-*KCNH2*-qF | 5’CATCTGCGTCATGCTCATTGGC3’ |
| h-*KCNH2*-qR | 5’TCTGGTGGAAGCGGATGAACTC3’ |
| h-*ETS2*-qF | 5’ACTCCGCCAACTGTGAATTGCC3’ |
| h-*ETS2*-qR | 5’CCACTGGCATACCTGTTGCTCA3’ |
| h-*MYOCD*-qF | 5’GCAACACCGATTCAGCTACCTAG3’ |
| h-*MYOCD*-qR | 5’GGTATTGCTCAGTGGCGTTGAAG3’ |
| h-*MEF2A*-qF | 5’CAAGGGCATGATGCCTCCACTA3’ |
| h-*MEF2A*-qR | 5’GCTGAGTACACAAGTCCTTGCG3’ |
| h-*DPF3*-qF | 5’CCTCTCAGGAAGACCACGACAA3’ |
| h-*DPF3*-qR | 5’CAGGTGAGTGTGAGCATAGTGG3’ |

**Table S2 Transcription factors regulated by HAND1 and HAND2 in *H1*/*H2*-dKO downregulated genes.**

| **GeneID** | **Gene** | ***H1*/*H2*-dKO vs WT**  **log2FoldChange** | **p.adj** |
| --- | --- | --- | --- |
| ENSG00000184828 | *ZBTB7C* | -5.58049 | 4.77E-84 |
| ENSG00000105880 | *DLX5* | -5.54548 | 4.99E-05 |
| ENSG00000125813 | *PAX1* | -5.04824 | 6.01E-17 |
| ENSG00000008196 | *TFAP2B* | -4.87438 | 0.00084 |
| ENSG00000083307 | *GRHL2* | -4.44675 | 3.81E-28 |
| ENSG00000113196 | *HAND1* | -4.44561 | 0 |
| ENSG00000275410 | *HNF1B* | -4.29706 | 2.57E-22 |
| ENSG00000087510 | *TFAP2C* | -4.28501 | 4.88E-06 |
| ENSG00000075426 | *FOSL2* | -3.81938 | 1.88E-146 |
| ENSG00000115112 | *TFCP2L1* | -3.76646 | 2.97E-05 |
| ENSG00000174576 | *NPAS4* | -3.71393 | 8.89E-176 |
| ENSG00000187098 | *MITF* | -3.5693 | 2.14E-247 |
| ENSG00000162761 | *LMX1A* | -3.41758 | 0.028563 |
| ENSG00000125816 | *NKX2-4* | -3.26085 | 0.003754 |
| ENSG00000141052 | *MYOCD* | -3.1769 | 3.29E-195 |
| ENSG00000116132 | *PRRX1* | -2.82994 | 0.020725 |
| ENSG00000101096 | *NFATC2* | -2.71118 | 2.24E-129 |
| ENSG00000116016 | *EPAS1* | -2.66262 | 6.87E-29 |
| ENSG00000109819 | *PPARGC1A* | -2.56432 | 8.02E-139 |
| ENSG00000185811 | *IKZF1* | -2.48116 | 1.52E-06 |
| ENSG00000205683 | *DPF3* | -2.43804 | 2.50E-170 |
| ENSG00000175329 | *ISX* | -2.36447 | 0.000112 |
| ENSG00000103460 | *TOX3* | -2.06692 | 2.20E-10 |
| ENSG00000152784 | *PRDM8* | -1.98629 | 2.70E-36 |
| ENSG00000157613 | *CREB3L1* | -1.90737 | 5.24E-52 |
| ENSG00000125740 | *FOSB* | -1.88745 | 2.60E-197 |
| ENSG00000089225 | *TBX5* | -1.87272 | 2.98E-65 |
| ENSG00000162772 | *ATF3* | -1.83203 | 8.16E-117 |
| ENSG00000114315 | *HES1* | -1.75603 | 9.84E-127 |
| ENSG00000043039 | *BARX2* | -1.74718 | 0.00181 |
| ENSG00000151014 | *NOCT* | -1.73023 | 3.46E-22 |
| ENSG00000164649 | *CDCA7L* | -1.70289 | 8.11E-90 |
| ENSG00000139083 | *ETV6* | -1.66591 | 1.69E-70 |
| ENSG00000256463 | *SALL3* | -1.62735 | 2.73E-29 |
| ENSG00000169594 | *BNC1* | -1.62452 | 2.36E-20 |
| ENSG00000078900 | *TP73* | -1.6164 | 0.009542 |
| ENSG00000079102 | *RUNX1T1* | -1.58153 | 2.34E-54 |
| ENSG00000198517 | *MAFK* | -1.5749 | 3.86E-44 |
| ENSG00000138795 | *LEF1* | -1.54334 | 1.00E-48 |
| ENSG00000157557 | *ETS2* | -1.509 | 1.92E-95 |
| ENSG00000067082 | *KLF6* | -1.50691 | 1.37E-98 |
| ENSG00000116604 | *MEF2D* | -1.47437 | 3.55E-145 |
| ENSG00000124782 | *RREB1* | -1.44402 | 4.39E-48 |
| ENSG00000121075 | *TBX4* | -1.40546 | 0.020449 |
| ENSG00000068305 | *MEF2A* | -1.39056 | 6.61E-51 |
| ENSG00000135111 | *TBX3* | -1.32924 | 3.37E-84 |
| ENSG00000187079 | *TEAD1* | -1.31568 | 9.34E-24 |
| ENSG00000146676 | *PURB* | -1.30898 | 8.00E-38 |
| ENSG00000183072 | *NKX2-5* | -1.30509 | 5.97E-55 |
| ENSG00000198146 | *ZNF770* | -1.26489 | 2.47E-21 |
| ENSG00000213020 | *ZNF611* | -1.21436 | 3.76E-28 |
| ENSG00000172493 | *AFF1* | -1.19241 | 3.75E-19 |
| ENSG00000185774 | *KCNIP4* | -1.19241 | 3.67E-09 |
| ENSG00000169155 | *ZBTB43* | -1.17588 | 7.03E-26 |
| ENSG00000134250 | *NOTCH2* | -1.14926 | 1.11E-18 |
| ENSG00000060237 | *WNK1* | -1.14698 | 2.10E-19 |
| ENSG00000122779 | *TRIM24* | -1.12103 | 3.59E-67 |
| ENSG00000101665 | *SMAD7* | -1.12023 | 2.49E-91 |
| ENSG00000124831 | *LRRFIP1* | -1.11507 | 2.14E-81 |
| ENSG00000169926 | *KLF13* | -1.05349 | 8.41E-45 |
| ENSG00000172216 | *CEBPB* | -1.00921 | 1.12E-12 |
