# Supplementary material for: HAND factors regulate cardiac lineage commitment and differentiation from human pluripotent stem cells
Source: Stem Cell Res Ther. 2024 Feb 5;15:31. doi: 10.1186/s13287-024-03649-9 (PMC10845658; doi:10.1186/s13287-024-03649-9)

**Additional file 8**

**Fig. S7: Full-length blots of Fig. 1H.**

Establishment of knockout cell lines. Western blot analysis of HAND1 and HAND2 in KO cell lines-derived day 10 cardiomyocytes.


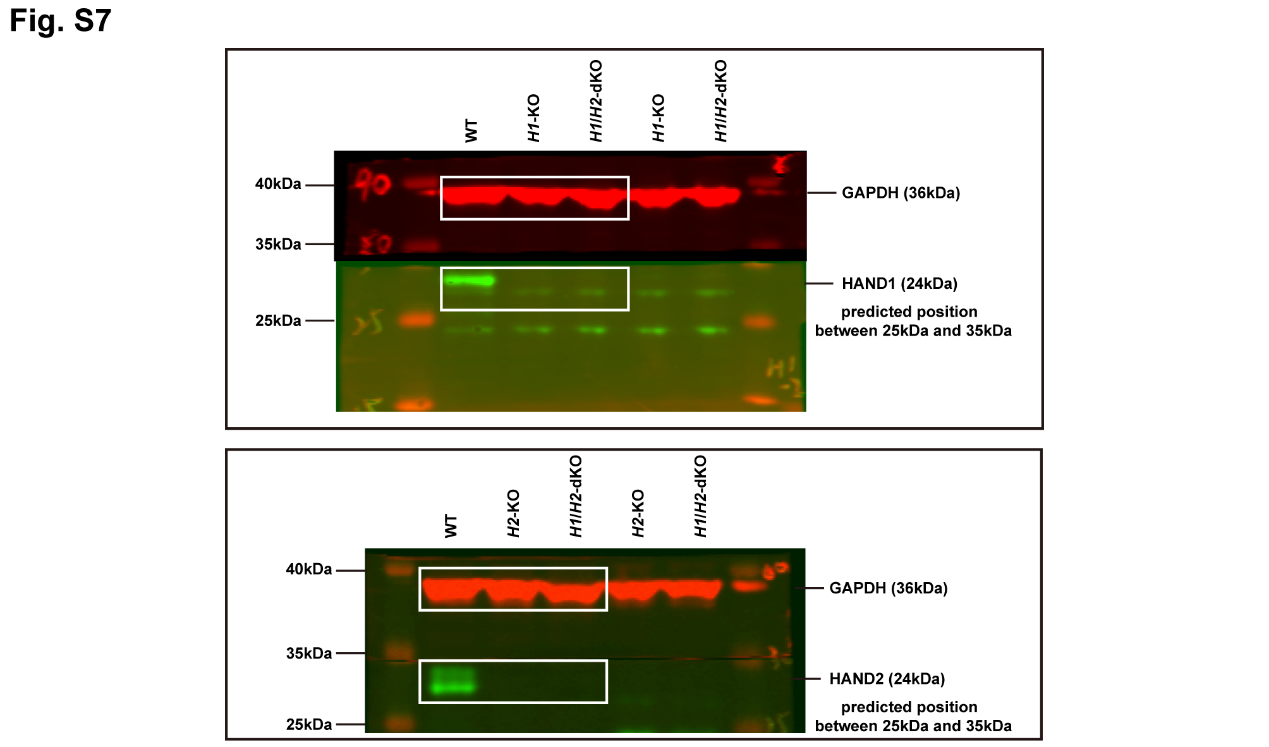


**Fig. S8: Full-length blots of Fig. 2F and Fig. S2H, J.**

Characteristics of *H1*-KO day 30 cardiomyocytes. Western blot analysis of CX43, MYL2, NR2F2 and SCN5A in *H1*-KO-derived day 30 cardiomyocytes.


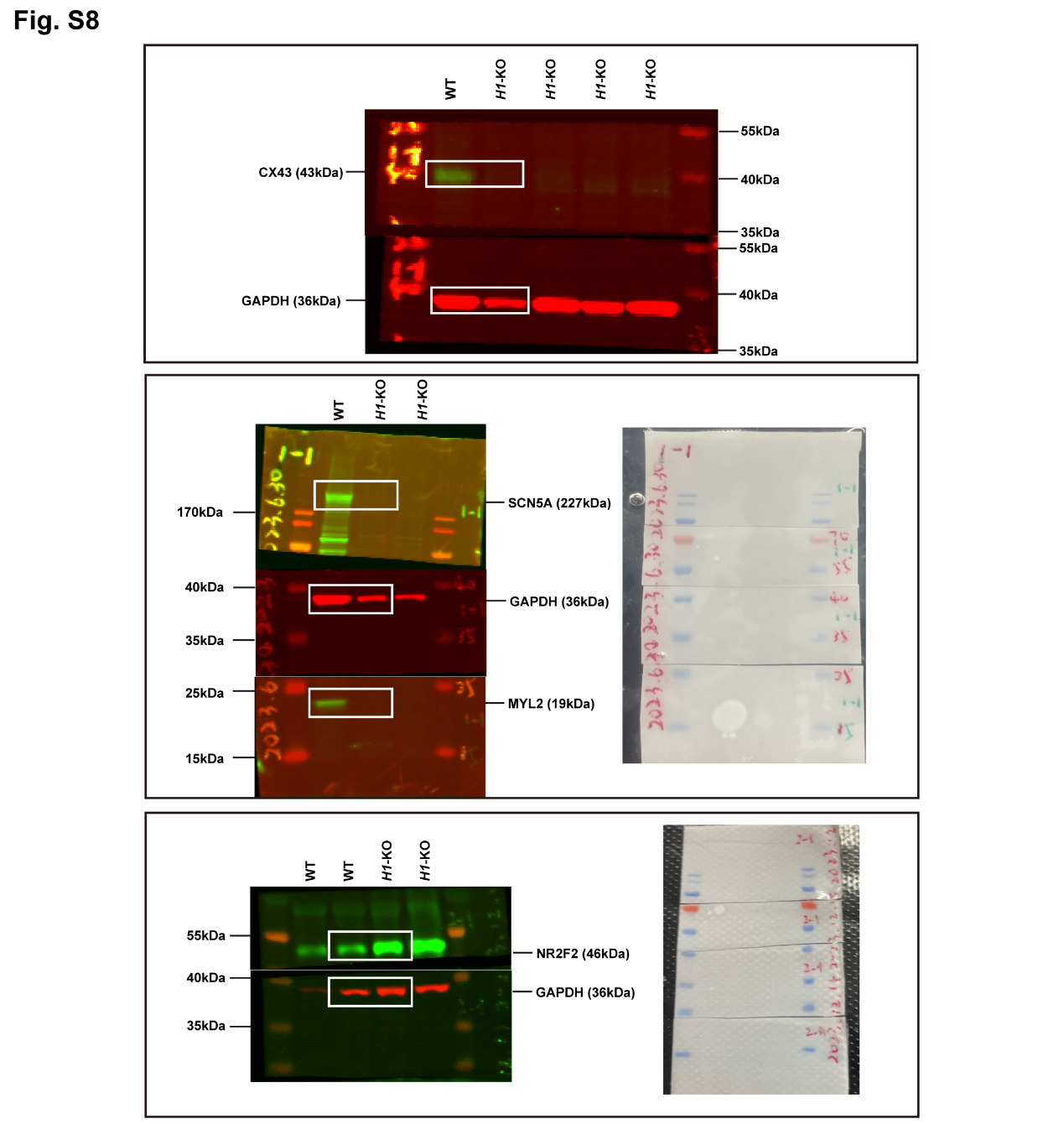


**Fig. S9: Full-length blots of Fig. 3F and Fig. S3G.**

Characteristics of *H2*-KO day 30 cardiomyocytes. Western blot analysis of MYL2 and NR2F2 in *H2*-KO-derived day 30 cardiomyocytes.


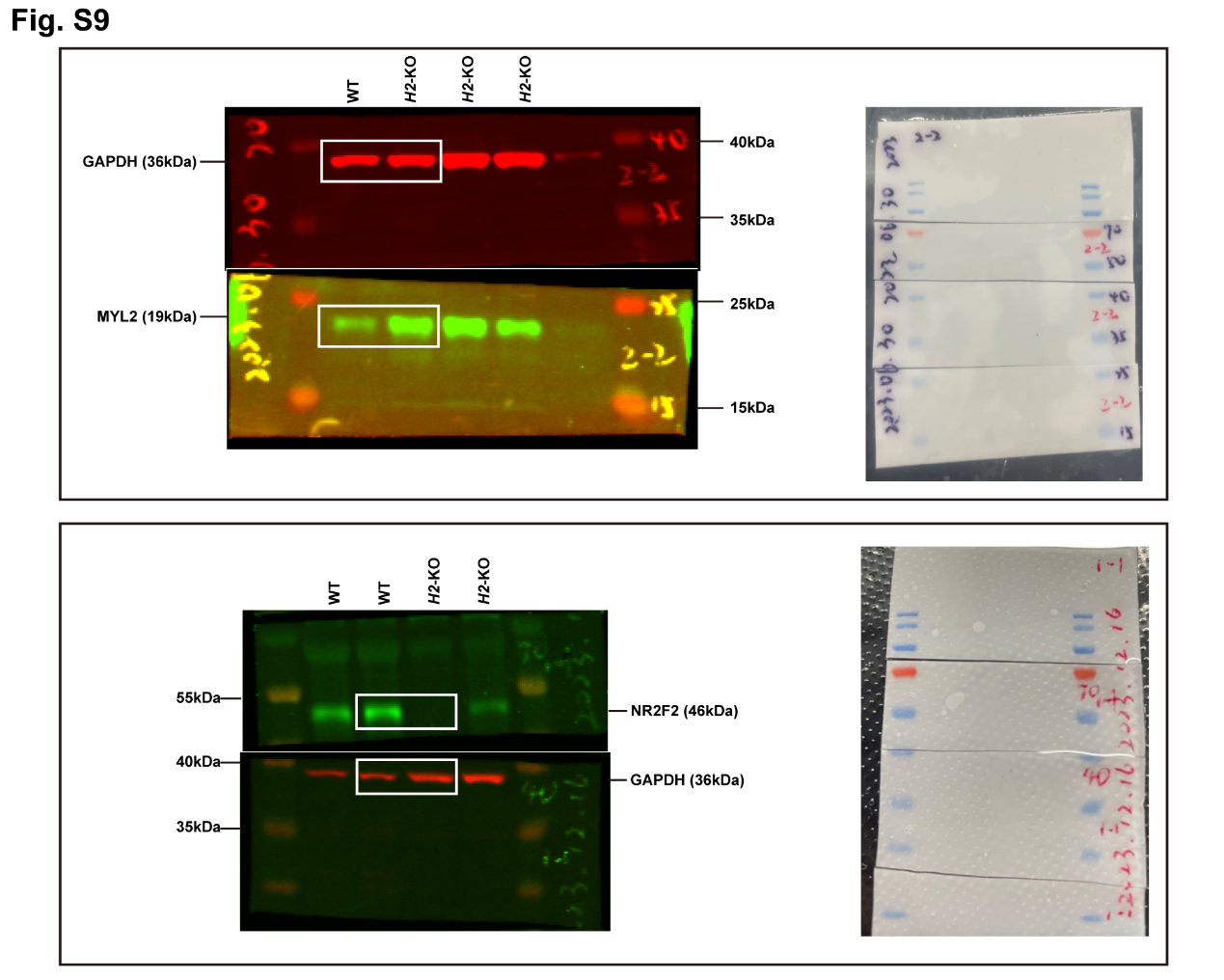


**Fig. S10: Full-length blots of Fig. 4F and Fig. S4B, C, J, L.**

Characteristics of *H1*/*H2*-dKO day 30 cardiomyocytes. Western blot analysis of CX43, MYL2, NR2F2 and SCN5A in *H1*/*H2*-dKO-derived day 30 cardiomyocytes.

Western blot analysis of HAND1 and HAND2 in *H2*-KO and *H1*-KO cells during early cardiomyocyte differentiation, respectively.


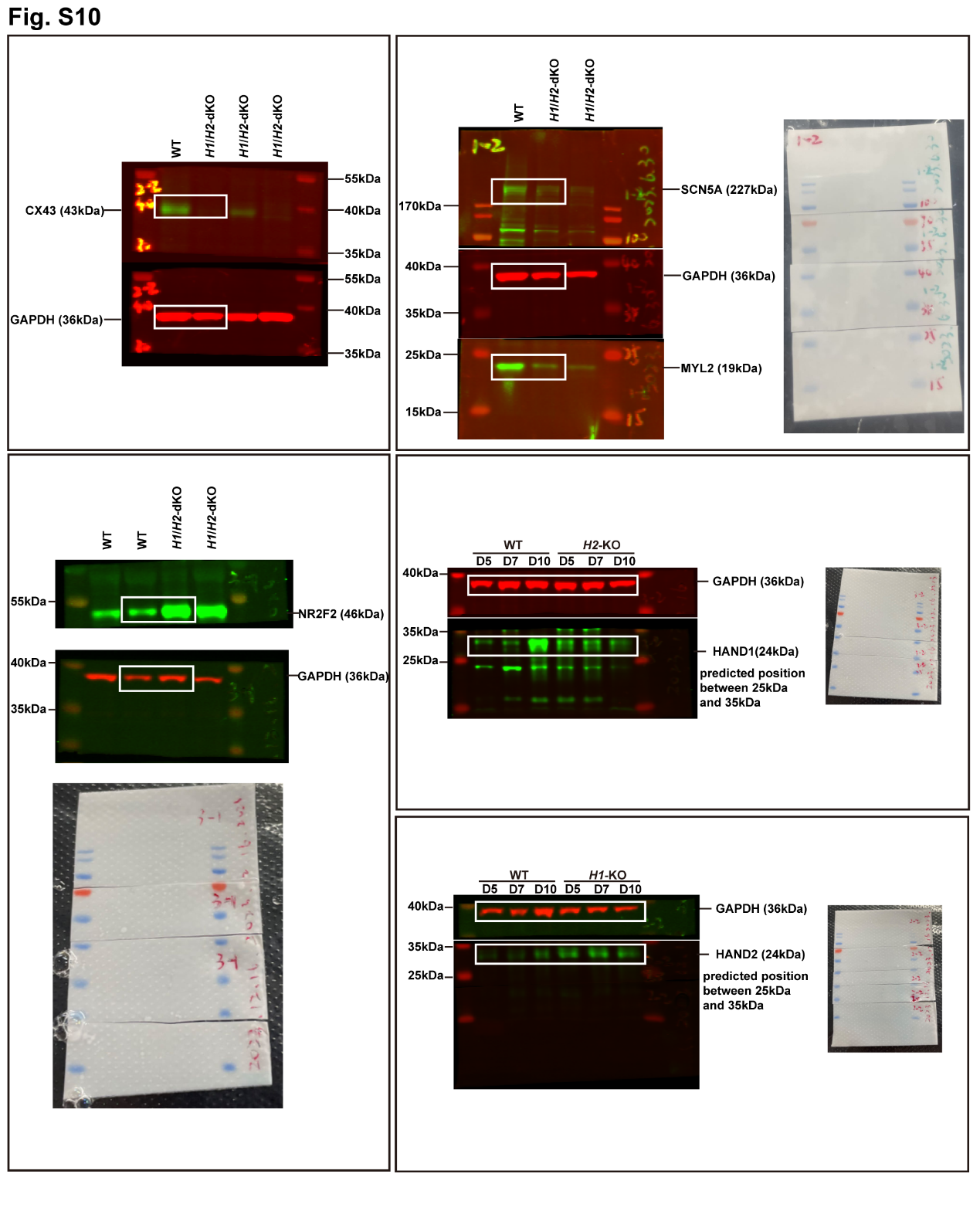


**Fig. S11: Full-length blots of Fig. 6D.**

Successful expression of TBX5 under DOX induction after 48 h. Western blot analysis of TBX5 in *TBX5*-OE cell lines (-DOX/+DOX) at differentiation day 5 and 10.


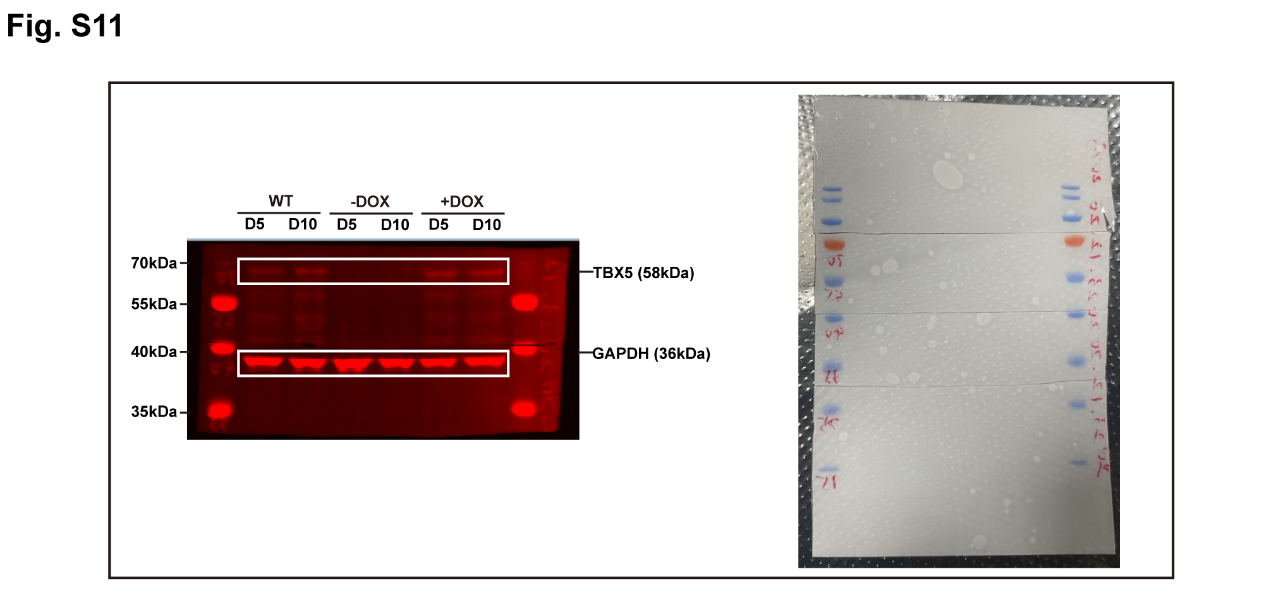

Supplement: Supplementary file 8 — Additional file 8. Fig. S7. Full-length blots of Fig. 1H. Western blot analysis of HAND1 and HAND2 expression in KO cell lines-derived day 10 cardiomyocytes. Fig. S8. Full-length blots of Fig. 2F and Fig. S2H, J. Western blot analysis of CX43, MYL2, NR2F2 and SCN5A expression in H1-KO-derived day 30 cardiomyocytes. Fig. S9. Full-length blots of Fig. 3F and Fig. S3G. Western blot analysis of MYL2 and NR2F2 expression in H2-KO-derived day 30 cardiomyocytes. Fig. S10. Full-length blots of Fig. 4F and Fig. S4B, C, J, L. Western blot analysis of CX43, MYL2, NR2F2 and SCN5A expression in H1/H2-dKO-derived day 30 cardiomyocytes. Western blot analysis of HAND1 and HAND2 expression in H2-KO and H1-KO cells, respectively. Fig. S11. Full-length blots of Fig. 6D. Western blot analysis of TBX5 expression in TBX5-OE cell lines (-DOX/+DOX) at differentiation days 5 and 10. [file 13287_2024_3649_MOESM8_ESM.docx]
